# Community-acquired *Staphylococcus aureus* skin and soft tissue infection risk assessment using hotspot analysis and risk maps: the case of California emergency departments

**DOI:** 10.1186/s12889-023-17336-6

**Published:** 2024-01-09

**Authors:** Brittany L. Morgan Bustamante, Laura Fejerman, Larissa May, Beatriz Martínez-López

**Affiliations:** 1 Public Health Sciences, School of Medicine, University of California, Davis, Davis, CA USA; 2 Center for Animal Disease Modeling and Surveillance, Department of Veterinary Medicine and Epidemiology, School of Veterinary Medicine, University of California, Davis, Davis, CA USA; 3 Emergency Medicine, School of Medicine, University of California, Davis, Davis, CA USA

**Keywords:** CA-MRSA, CA-MSSA, *Staphylococcus aureus*, Skin and soft tissue Infections, Geographic disparities, Spatial analysis, Place-based determinants, Hotspot analysis, Medical service study areas

## Abstract

**Background:**

Community-acquired *Staphylococcus aureus* (CA-*Sa*) skin and soft tissue infections (SSTIs) are historically associated with densely populated urban areas experiencing high poverty rates, intravenous drug use, and homelessness. However, the epidemiology of CA-Sa SSTIs in the United States has been poorly understood since the plateau of the Community-acquired Methicillin-resistant *Staphylococcus aureus* epidemic in 2010. This study examines the spatial variation of CA-Sa SSTIs in a large, geographically heterogeneous population and identifies neighborhood characteristics associated with increased infection risk.

**Methods:**

Using a unique neighborhood boundary, California Medical Service Study Areas, a hotspot analysis, and estimates of neighborhood infection risk ratios were conducted for all CA-Sa SSTIs presented in non-Federal California emergency departments between 2016 and 2019. A Bayesian *Poisson* regression model evaluated the association between neighborhood-level infection risk and population structure, neighborhood poverty rates, and being a healthcare shortage area.

**Results:**

Emergency departments in more rural and mountainous parts of California experienced a higher burden of CA-Sa SSTIs between 2016 and 2019. Neighborhoods with high infection rates were more likely to have a high percentage of adults living below the federal poverty level and be a designated healthcare shortage area. Measures of population structure were not associated with infection risk in California neighborhoods.

**Conclusions:**

Our results highlight a potential change in the epidemiology of CA-Sa SSTIs in California emergency departments. Future studies should investigate the CA-Sa burden in other geographies to identify whether this shift in epidemiology holds across other states and populations. Further, a more thorough evaluation of potential mechanisms for the clustering of infections seen across California neighborhoods is needed.

**Supplementary Information:**

The online version contains supplementary material available at 10.1186/s12889-023-17336-6.

## Background

*Staphylococcus aureus* is an opportunistic commensal bacterium that is the most common cause of skin and soft-tissue infections (SSTIs) in the United States [1, 2]. *S. aureus* has been endemic in the US healthcare setting for several decades and was once considered an isolated issue for hospitals [3]. However, starting in the mid-1990s, Methicillin-resistant *Staphylococcus aureus* (MRSA) began increasing in communities across the US [4]. This new strain, deemed community-acquired or community-associated MRSA (CA-MRSA), spread rapidly among patients with and without previous exposure to the healthcare environment [5]. By 2007, most major cities in the US had reported CA-MRSA cases [5]. CA-MRSA is now more predominant than healthcare-acquired MRSA [2] and has been an issue in densely populated urban areas with higher rates of homelessness, incarceration, intravenous drug use, and household crowding [1, 6–8]. CA-MRSA SSTIs are rarely fatal, but treatment failure ranges between 15 and 38% [9], resulting in complications such as hospitalization, surgery, bloodstream infections or bacteremia, and substantial patient morbidity [10]. Further, CA-MRSA SSTIs are a significant financial burden on healthcare systems [11].

Previous research shows that CA-MRSA infections vary geographically [12–15] and by neighborhood socioeconomic (SES) indicators [7, 16]. When comparing MRSA rates across global regions, the lowest prevalence has historically been in Scandinavia and Canada, and the highest prevalence has historically been in the United States, Latin America, Hong Kong, and Japan [17]. In the US, most studies evaluating the prevalence and epidemiology of CA-MRSA have been limited to cities or metropolitan areas [1, 4, 18–24]. In Atlanta, Georgia, a study on children identified higher rates of CA-MRSA in neighborhoods with a higher proportion of Black residents, household crowding, and children under four years of age [13]. In New York City, urban regions with higher CA-MRSA prevalence also had lower SES, more overcrowding, and high HIV prevalence [7, 25]. Studies conducted on only a micro- (single city) or macro-level (across countries) scale makes comparing disease burden between geographies or generalizing the results challenging.

For many years, CA-MRSA was a primary concern among clinicians and researchers. However, the proportion of community-acquired *S. aureus* (CA-Sa) SSTIs caused by CA-MRSA has decreased over the last decade in North America, Latin America, Europe, and Japan—while rates of community-acquired Methicillin-susceptible *S. aureus* (CA-MSSA) have increased [26]. CA-MSSA and CA-MRSA SSTI risk factors are similar, and outcomes differ slightly. CA-MSSA SSTIs have higher hospitalization rates [27], but there do not appear to be differences in the need for surgical drainage [27], treatment failure [27], mortality [27], or abscess size [28]. Since CA-MRSA mainly drove the increase in SSTIs observed in the US between 1997 and 2007, and the CA-MRSA epidemic plateaued in 2010, the epidemiology of SSTIs has been poorly understood since then [29]. To our knowledge, the most recent epidemiological assessment of SSTIs in a US outpatient setting is from 2015 [30] and 2016 for recurrent SSTIs [31], warranting an updated understanding of the current burden of CA-Sa SSTIs.

This paper addressed this epidemiological gap by estimating CA-Sa SSTI risk in an underutilized geographic analysis unit, California’s Medical Service Study Areas (MSSAs). MSSAs are the defined geographic analysis unit for California’s Department of Healthcare Access and Information (HCAI) and are California’s accepted Rational Service Area for medical service [32]. MSSAs incorporate total population, socioeconomic, and demographic data provided by the US Census combined with healthcare services and availability to define area boundaries that maximize homogeneity in the social and structural environment. MSSAs represent where individuals within the area reasonably seek healthcare services, accounting for commuting patterns and physical barriers like highways, mountains, and bodies of water [33]. MSSAs comprise one or more complete census tracts – small, relatively permanent statistical subdivisions of a county or statistically equivalent entity [34] – and do not cross county lines. They offer spatial resolution at a finer scale than the county, and while not as granular as census tract, the boundaries of MSSAs are less arbitrary.

This study aimed to characterize the spatial variation in CA-Sa SSTI rates across a large, heterogeneous population and to estimate the infection risk ratio (IRR) in California MSSAs. To the best of our knowledge, MSSA is a novel neighborhood definition for identifying infection clusters, modeling small-area IRRs, spatial variation, and incidence of CA-Sa SSTIs in California. Combined, this serves as an update to our understanding of the epidemiology of infection and identifies areas of high infection burden that can be informative to public health practitioners and policymakers.

## Methods

### Data used

Patient health data was gathered from nonpublic emergency department (ED) discharge data from HCAI between 2016 and 2019 [35]. HCAI collects yearly patient demographic data, clinical visit details, and payer and facility information from every non-federal hospital licensed to provide emergency medical services in California. This data constitutes a comprehensive record of ED visits to non-federal general acute care hospitals across the state. In California, ED use has increased considerably since the expansion of Medicaid in 2014 [36]. In 2005, there were approximately 10 million visits to the ED. ED visits increased by more than 40% within ten years to 14.5 million. When adjusted for population growth during the same period, the increase is still sizeable at 31% [36]. The increased usage and reliance on EDs in California, combined with the large percentage of reported CA-Sa SSTIs being treated in EDs [37], make the HCAI data a promising dataset for measuring population-level incidence of CA-Sa SSTIs.

The geographic unit for this analysis was California MSSA, which we used to define an individual’s neighborhood. However, patient residential location data from HCAI is available only at the ZIP code level, administrative boundaries the US Postal Service developed to deliver mail. ZIP codes are not recommended as the geographic analysis unit for research because they are not representative of human behavior, nor do they coincide with municipality boundaries, and, when used in data analysis, they can mask insights [32]. Using ZIP codes as a unit of analysis in public health studies has been criticized [38], and improving the ability to crosswalk ZIP codes to more meaningful boundaries has recently been a focus for the US Department of Housing and Urban Development [39, 40]. We crosswalked—translating values from one schema to another—ZIP codes to MSSAs using the yearly US Postal Service ZIP code to census tract crosswalk files from the US Department of Housing and Urban Development. These files are derived from the quarterly vacancy data from the United States Postal Service [41].

### ZIP code to census tract & MSSA

Crosswalking ZIP codes directly to MSSA requires creating population-weighted centroids for each ZIP code area and overlapping those with each MSSA [32]. However, population-weighted centroids use single points to represent polygons and allocate entire ZIP codes to the MSSA that contains the majority of the population. This method works well for most ZIP codes in California since over 80% are entirely or mostly (> 90% of their area) contained within a single MSSA [32]. However, for the 19% of ZIP codes that cross an MSSA boundary, using an allocation method based on the residential population density of the census tracts falling within a ZIP code allowed us to consider the spatial distribution of a population. It also allowed us to account for the unequal distribution across space if we needed to allocate one ZIP code to multiple MSSAs.

**Fig. 1:**
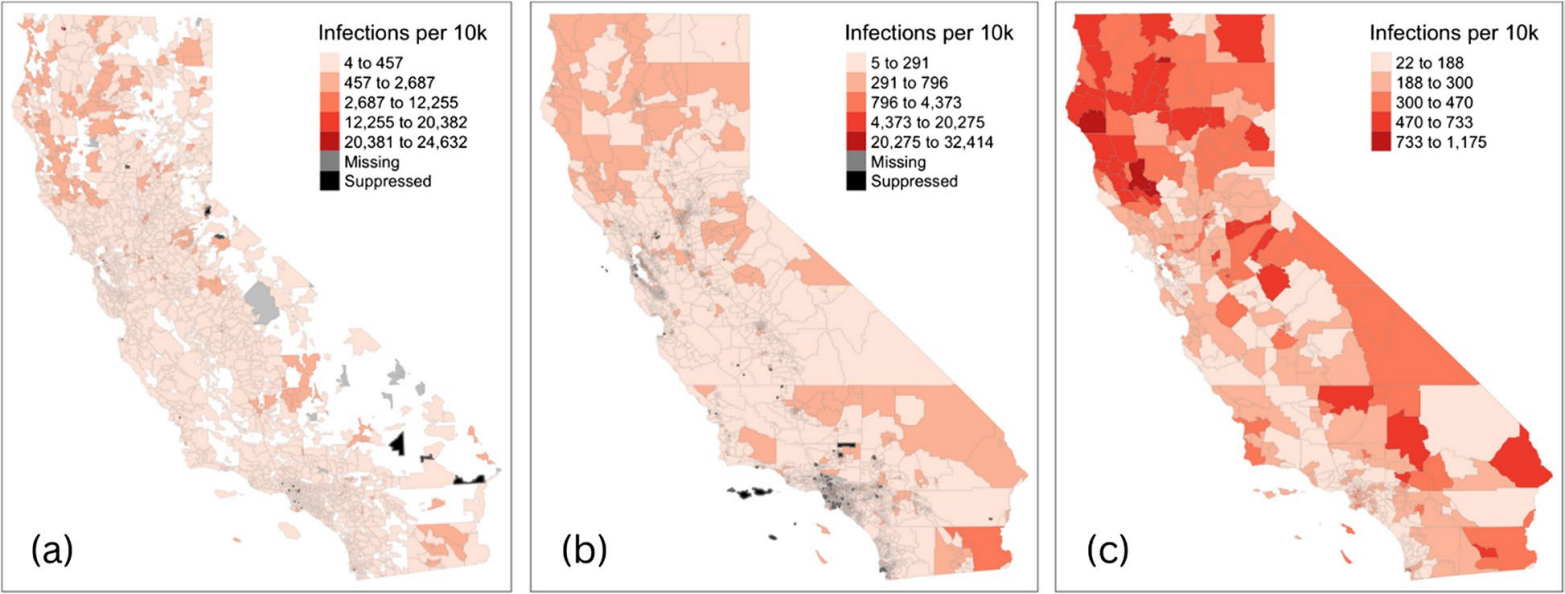
Geographic distribution of CA-Sa SSTI rates presenting in emergency departments in California between 2016-2019. Panels are as follows: **a** infection rates per 10,000 residents using the originally reported patient ZIP code (proxied by ZIP code tabulation area) as the geographic analysis unit (*n* = 1,769); **b** infection rates per 10,000 residents using randomly assigned census tracts based on residential density within patient ZIP codes as the geographic analysis unit (*n*=7,984); and **c** infection rates per 10,000 residents using the crosswalked Medical Service Study Area as the geographic analysis unit (*n*=542). Geographies where the estimated population was less than 20 residents have been suppressed. All variables were classified using natural breaks

 Patients with missing ZIP codes or ZIP codes outside California were removed. Patients were randomly assigned a census tract within their ZIP code so that the proportion of study patients in each census tract within a ZIP code equaled the reported proportions by the US Department of Housing and Urban Development [41]. That is, the probability of being assigned a census tract corresponded to the percent of residential addresses in each census tract with which the ZIP code overlapped. Then, census tracts were combined, and the data was aggregated to the appropriate MSSA. Since individuals were assigned a census tract that overlapped with their documented ZIP code, proportionate to residential density, and several census tracts make up an MSSA, individuals who may have been assigned a census tract adjacent to the one in which they live would have still been assigned to the appropriate MSSA in the second step of the crosswalk. To evaluate how sensitive the geographic patterns of CA-Sa SSTIs were to how patients were allocated, we visually compared choropleth maps using a *jenks* classifier [42] with ZIP code tabulation areas (ZCTA) (Fig. 1a), the randomly assigned census tracts (Fig. 1b), and the crosswalked MSSAs as the geographic unit (Fig. 1c).

### Identifying CA-Sa cases

*S. aureus* is the predominant cause of subcutaneous abscesses and the second most common infection-causing microbe in cellulitis cases, *Streptococcus pyogenes* being the first [43]. Misdiagnosis of these entities is common, as are coding errors between the two [44]. Further, before implementing the International Classification of Diseases, Tenth Revision (ICD-10) codes, abscess diagnoses were combined with cellulitis as a single diagnostic code. For these reasons, CA-Sa SSTI cases were defined as those treated in the ED during the study period with a principal diagnosis (ICD-10) of impetigo (L01), cutaneous abscess, furuncle and carbuncle (L02), cellulitis and acute lymphangitis (L03), erysipelas (A46), other local infections of skin and subcutaneous tissue (L08.89), and other specified disease of hair and hair follicles (L73.8). We used these codes as a proxy for CA-Sa SSTI since CA-Sa is more likely to cause these types of infections, and these codes were most often used in previous studies of CA-Sa SSTIs [1, 45, 46].

Encounters indicative of a repeat visit for the same infection (occurring within ten days after the first encounter) were identified using record linkage number matching and excluded. Ten days was chosen as the cutoff because randomized control trials have used this period to define the clinical cure of SSTI following antibiotic treatment [47]. Record linkage numbers are derived from social security numbers. Individuals without a social security number will not have a record linkage number in the dataset. For the individuals without a record linkage number (*n* = 90,228; <1% of the total sample), we matched repeat visits on patient ZIP code, sex, age at service, racial/ethnic group, and payer information. Repeat encounters were evaluated across all EDs; an encounter did not have to occur at the same facility as the first encounter to be excluded.

### Standardized Infection Ratio (SIR) mapping

The spatial distribution of CA-Sa SSTI rates per 10,000 residents in each neighborhood was visually evaluated using choropleth maps. To create SIR maps, the age-stratified expected number of CA-Sa cases in each California neighborhood was calculated, and SIRs were developed according to Eq. 1. Expected cases were calculated by using the age-stratified infection rates from the entire California population and applying those rates to the population age distribution of the neighborhood (i.e., multiplying the overall age-stratified incidence rate in California with the number of individuals in each age stratum living in each neighborhood) using the *SpatialEpi* package in R [48]. The SIRs were mapped in a choropleth map to visualize the observed and expected infection counts; SIR = 1 indicates observed cases are the same as expected; SIR > 1 indicates observed cases are higher than expected; and SIR < 1 indicates observed cases are lower than expected.

1 $${SIR}_{i}= \frac{{Y}_{i}}{{E}_{i}}$$

#### Equation 1: Standardized Infection Ratio equation (SIR)

*Y*_*i*_ *is the number of observed cases in neighborhood i; E*_*i*_ *is the number of expected cases in that same neighborhood*.

### Hotspot analysis

Due to the polygon nature of the data, queen’s adjacency was used to define neighbor connectivity. Queen adjacency defines neighbors as any areas sharing a line segment (border) or a point (a vertex). One neighborhood, a small island off the west coast of Los Angeles with no neighbors, was removed from the analysis. Neighbor weights were calculated using row standardization [48]. Apparent clustering identified in the choropleth map was explored using a Moran scatterplot, which plots the standardized infection rates on the x-axis and the standardized average infection rate of one’s neighbors (known as the spatial lag) on the y-axis [49].

A global measure of spatial autocorrelation (Moran’s I) was calculated to quantify an objective measure of the degree to which similar infection rates cluster. Monte-Carlo simulations of Moran’s I statistic with 1,000 runs were used to confirm results from the measure of global spatial autocorrelation. Three versions of Local Indicators of Spatial Autocorrelation were calculated to identify where clusters are located. The Z-scores from Getis-Ord Gi, Gi*, and Local Moran’s I were mapped and visually compared to gain insight into the location of areas with comparatively high or low associations with neighboring values (i.e., hot or cold spots). Local spatial autocorrelation analyses were conducted in R with the package *spdep* [49]. The classification of neighborhoods identified as significant in the local spatial autocorrelation analysis was based on the Local Moran’s I p-value and designated as a high-high cluster, low-low cluster, and high-low or low-high spatial outlier. These categories identifying the directionality of clusters and outliers were mapped and visually inspected.

### Spatial regression and risk map

A model-based approach was used to evaluate the presence of spatial autocorrelation, smooth over extreme values, and better estimate IRR in small areas by borrowing information from geographic neighbors. The model-based approach was also used to evaluate the potential associative factors population structure, area-level poverty rates, and healthcare service accessibility. Population structure is a logical starting place when evaluating spatial variation in disease outcomes, as it often represents background factors associated with differences in health between populations [50]. Area-level poverty and healthcare accessibility were evaluated due to documented associations with CA-Sa and poorer health outcomes more broadly [7, 16, 51, 52]. Population structure was characterized by the percentage of working-age (18–64 years) individuals, the percentage of individuals identifying as a race/ethnicity other than non-Hispanic white, and an urban/rural indicator. Area-level poverty was characterized as the percentage of adults in the neighborhood living below 100% of the federal poverty level (FPL). Healthcare service accessibility was proxied by a binary variable indicating whether a neighborhood was a primary healthcare shortage area (HCSA). These variables were informed by the US Census and are included in the HCAI publicly available MSSA dataset [53].

Using *R-INLA*, we built a Besag-York-Mollie model using a *Poisson* distribution to estimate the IRR, ${\theta }_{i}$, in neighborhoods $i = 1,\dots ,n$ (Eq. 2) [54]. The default log-gamma prior with parameters (1, 0.00005) was used, and the model was scaled, making the generalized variance equal to one. Scaling is recommended for Intrinsic Gaussian Markov random field models, which have a scaled precision matrix reflecting the neighborhood structure of the model. Scaling assigns the same fixed hyperprior to the precision parameters of all Intrinsic Gaussian Markov random fields in the model, making the precision parameter of models with different conditional autoregressive priors comparable [55, 56]. The resulting IRRs and lower/upper limits of the 95% credible intervals (CrI) were mapped.

2 $$Y_i\vert\theta_i\sim Poisson\left(E_i\times\theta_i\right)\;\log\left(\theta_i\right)=\beta_0\times\beta_1\ast Age+\beta_2\ast\;RE_i+\beta_3\ast Rural_i+\beta_4\ast\;Poverty_i+\beta_5\ast\;HCSA_i\ast+u_i+v_i$$

#### Equation 2: R-INLA model for neighborhood Infection risk ratio calculation

- ${Y}_{i}$: number of observed cases in neighborhood *i*.
- ${E}_{i}$: number of expected cases in neighborhood *i* (offset).
- ${\theta }_{i}$: infection risk ratio in neighborhood *i*.
- $${\beta }_{0}$$: intercept
- ${\beta }_{1}:$ coefficient for the percent of individuals working age (18–64 years) in neighborhood *i*.
- ${\beta }_{2}:$ coefficient for the percent of adults identifying as a race/ethnicity other than non-Hispanic white in neighborhood *i*.
- ${\beta }_{3}:$ coefficient for a binary indicator of neighborhood *i* being rural.
- ${\beta }_{4}:$ coefficient for the percent of adults living below the federal poverty level in neighborhood *i*.
- ${\beta }_{5}:$ coefficient for a binary indicator of neighborhood *i* being an HCSA.
- ${u}_{i}$: structured spatial effect to account for the spatial dependence between neighborhoods (i.e., neighborhoods sharing a boundary can influence the risk amongst themselves)
- ${v}_{i}$: unstructured spatial effect to account for potential independence among the neighborhoods (i.e., neighborhoods that share a boundary may not influence the risk amongst themselves)

We rescaled the percent variables (working age, race/ethnicity other than non-Hispanic white, and poverty) to represent a 10% change by dividing the original percentage by 10. To evaluate the influence of the default log-gamma prior, we defined four different priors for the standard deviation: a half-normal, a half-Cauchy, a half-t, and an improper flat prior [57]. We also evaluated the Penalized Complexity prior, which penalizes departure from the base model [58]. The R code accompanying this analysis can be found in the Supplementary material.

## Results

A total of 977,968 encounters (2.46% of the total sample) were removed from the study because the individual resided outside California or had a missing ZIP code. ICD-10 codes indicative of a principal diagnosis of CA-Sa SSTI were identified in 844,692 case-patient visits. The majority of these were coded as ‘cellulitis and acute lymphangitis’ (63%). Most case patients had public insurance (68%), and most self-identified as male (55%). A complete demographic overview of case patients compared to the total sample is presented in Table 1.

**Table 1:** Demographics of individuals in California emergency department with principal diagnosis code of CA-Sa, 2016–2019 (*n* = 844,692 cases; *n* = 38,090,296 total sample)

	Cases		Total Sample	
Variable	n	%	n	%
*Sex*				
Male	465,350	55%	16,323,176	42.9%
Female	379,303	45%	21,765,363	57.1%
Missing	39	< 1%	1,757	< 1%
*Insurance*				
Public	575,449	68%	24,616,861	64.6%
Private	191,367	23%	10,606,963	27.8%
Uninsured	77,537	9%	2,809,905	7.4%
Missing	339	< 1%	56,567	< 1%
*Age*				
Median	45 years		44 years	
18–34	277,296	33%	12,941,938	34%
35–64	440,765	52%	17,289,610	45.4%
65+	126,631	15%	7,858,748	21.6%
*Race/Ethnicity*				
NH-White	390,419	46.2%	15,130,737	39.7%
NH-Black	87,431	10.4%	4,426,486	11.6%
NH-Asian	32,737	3.9%	2,324,293	6.1%
NH-Islander	24,349	2.9%	1,237,952	3.3%
NH-AIAN	5,195	< 1%	177,545	< 1%
Hispanic	286,243	33.9%	13,867,895	36.4%
Unknown	8,838	1.1%	436,942	1.1%
NH-Other	9,480	1.1%	488,446	1.3%
*Principal Diagnosis*				
Cellulitis and acute lymphangitis	543,162	62.6%		
Erysipelas	1,090	< 1%		
Cutaneous abscess, furuncle and carbuncle	307,219	35.4%		
Impetigo	10,911	1.3%		
Other local infections of skin and subcutaneous tissue	3,717	< 1%		
Other specified disease of hair and hair follicles	2,178	< 1%		

**Fig. 2:**
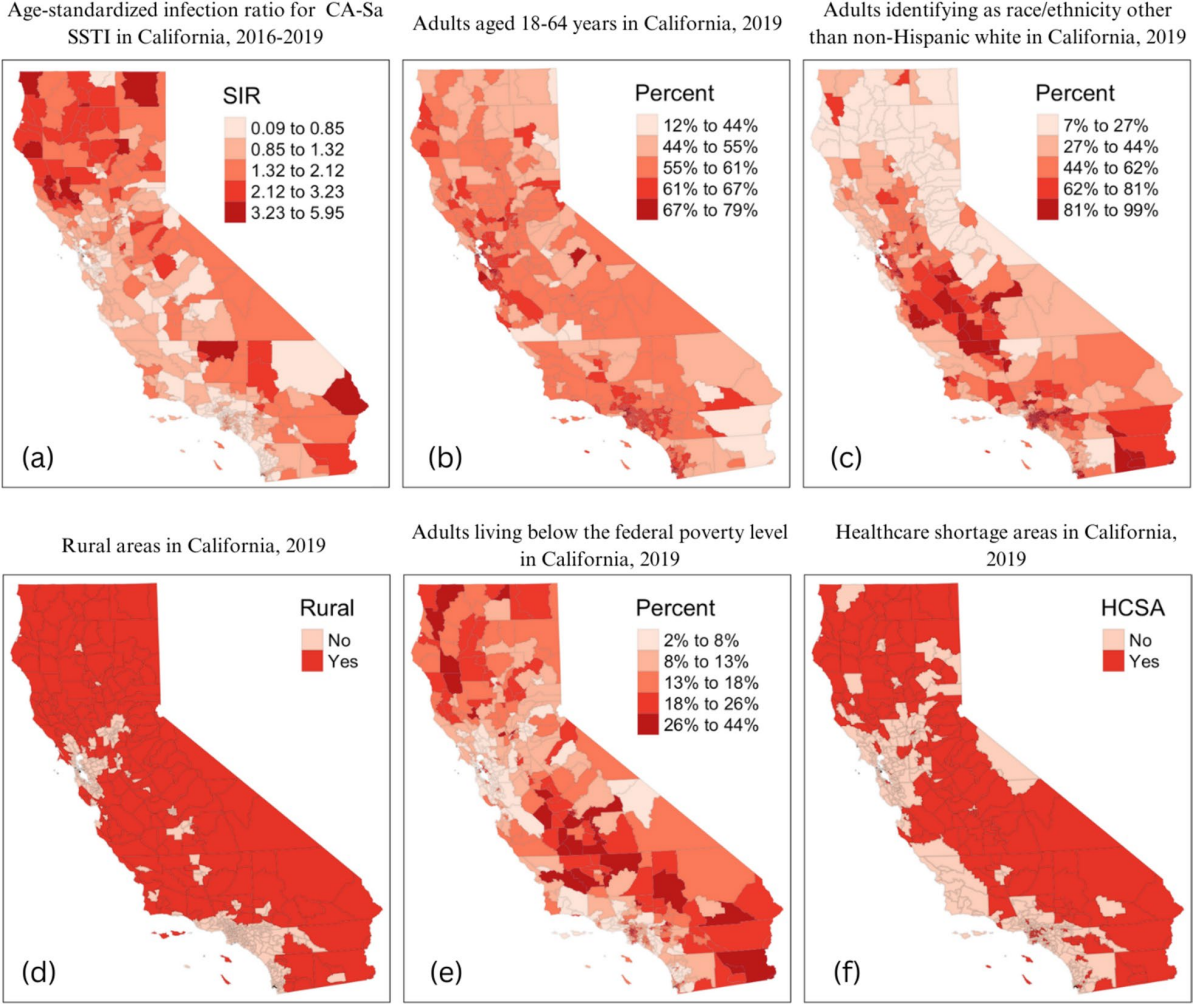
Distribution of SIRs for CA-Sa SSTIs and potential associative factors in California neighborhoods, 2016-2019 (*n *= 542). Panels are as follows: **a** Age-Standardized Infection Ratio (SIR) for CA-Sa SSTI; **b** Percentage of working age (aged 18-64) adults; **c** Percentage of adults (aged 18 and over) identifying as a race/ethnicity other than non-Hispanic white; **d** Rural areas; **e** Percentage of adults living below the federal poverty level; **f** Healthcare shortage areas (HCSA). All variables were classified using natural breaks

Infection rates consistently appeared highest in more rural, northwest parts of California in maps using the patient’s ZCTA (Fig. 1a), their randomly assigned census tract (Fig. 1b), and their crosswalked neighborhood (MSSA) (Fig. 1c). A map of the SIRs across neighborhoods confirmed this visual observation (Fig. 2a). High SIRs were seen in rural, mountainous areas of the state. Pockets of high rates were also dispersed around the southeast part of the state. Across the neighborhoods, working-age adults accounted for 12-78.7% of the total population. The highest percentages of working-age adults were in neighborhoods in and around major California cities (Fig. 2b). The highest concentration of individuals identifying as a race/ethnicity other than non-Hispanic white was found in California’s Central Valley and neighborhoods in the southern part of the state. Most residents of northern California neighborhoods identified as non-Hispanic white (Fig. 2c). While only 41% of neighborhoods (*n* = 222) were classified as rural or frontier, this equated to most of California’s land area and overlapped with high SIR areas (Fig. 2d). Several neighborhoods with high SIRs also had a high percentage of adults living below the FPL (Fig. 2e). Neighborhoods that were HCSAs were concentrated in parts of northern, central, and southeastern California (Fig. 2f). Slightly less than half (*n* = 227; 41.9%) of all neighborhoods in the state were HCSAs. However, those areas visually overlap with high SIR areas.

### Hotspot analysis

**Fig. 3:**
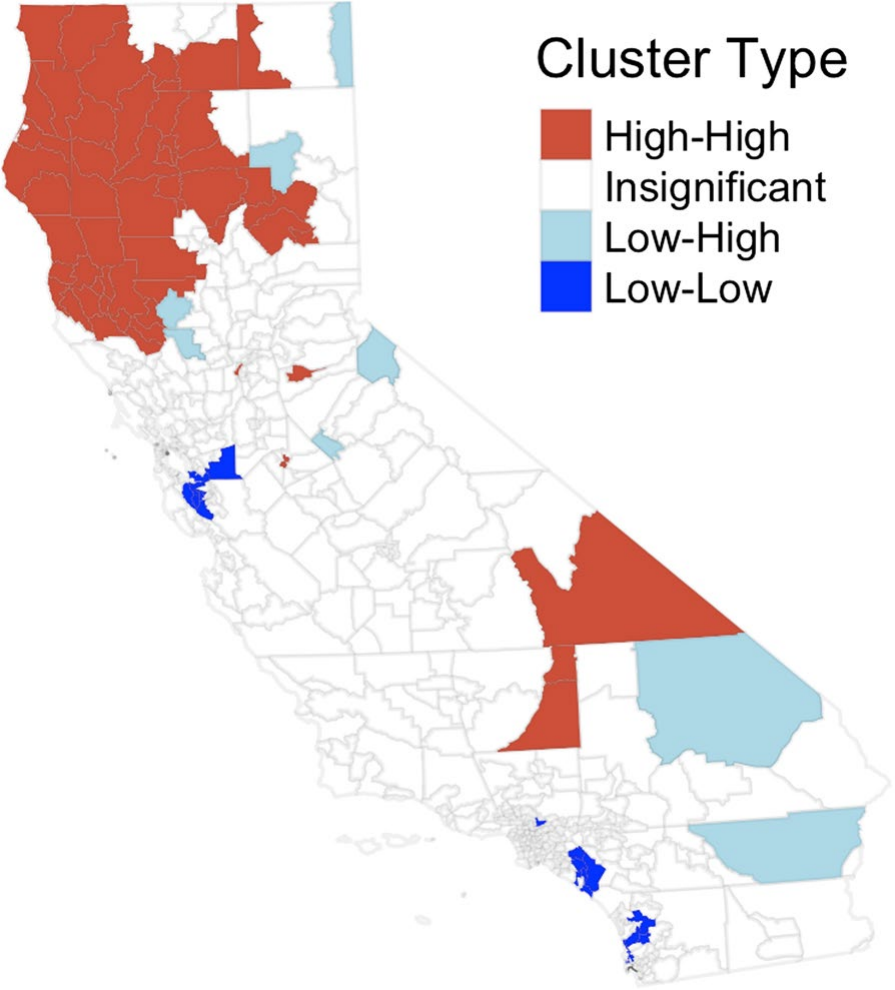
Hotspots of CA-Sa SSTI rates between 2016–2019 in California neighborhoods (*n* = 541)

Several neighborhoods in the northwest part of the state had high SIRs. Notably, Lucerne/Nice/Upper Lake neighborhood (SIR = 4.56) and Dunsmuir neighborhood (SIR = 4.35). Moran’s I for global spatial autocorrelation was statistically significant (Moran’s I: 0.497, *p* < 0.01). Tests for local spatial autocorrelation indicated significant clustering in the northwest and southeastern parts of the state, with a few clusters of low infection rates scattered throughout the rest of California (Fig. 3). There were some outliers of neighborhoods with low infection rates surrounded by high-rate areas (noted by light blue in Fig. 3). However, there were no outlier neighborhoods with high infection rates surrounded by low infection rate areas. A complete list of clusters and outliers can be found in Table A1.

### Spatial regression and risk map

**Fig. 4:**
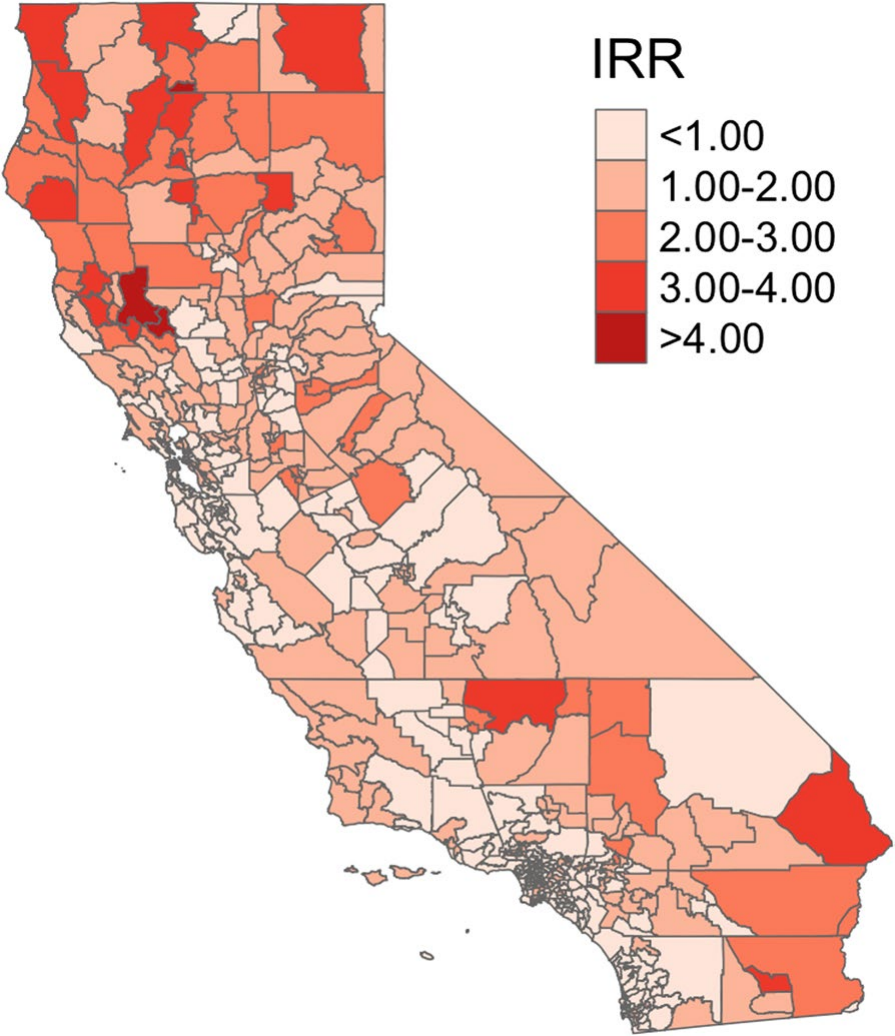
IRRs of CA-Sa SSTI in California neighborhoods between 2016-2019, after controlling for covariates (*n *= 541). Covariates include percentage of working age (aged 18-64) adults, percentage of adults (aged 18 and over) identifying as a race/ethnicity other than non-Hispanic white, rurality, percentage of adults living below the federal poverty level, and healthcare shortage areas

 The model-based approach exploring the geographic distribution for IRR estimates while controlling for population structure, the percent of adults living below the FPL, and whether the neighborhood was an HCSA supported the findings from the hotspot analysis. Areas with higher risk appeared in the northern, particularly the northwestern, and some eastern parts of the state. Table A2 lists the ten neighborhoods with the highest and lowest IRR for CA-Sa SSTIs, and Fig. 4 shows the geographic distribution of IRRs.

The posterior summary statistics from the R-INLA model are shown in Table 2. Population structure was only borderline significantly associated with CA-Sa SSTI. Neighborhoods with a higher percentage of working-age adults had slightly lower infection rates (IRR: 0.92; 95% CrI: 0.87, 0.97). Likewise, lower rates were associated with neighborhoods having a higher percentage of individuals identifying as a race/ethnicity other than non-Hispanic white (IRR: 0.95; 95% CrI: 0.93, 0.97). Whether a neighborhood was rural was not significantly associated with infection rates. A significant and positive association was observed between the percentage of adults living in poverty and neighborhood-level IRR of CA-Sa SSTI. The estimated IRR for a 10% increase in the percentage of adults living in poverty, holding the other variables in the model constant, was 1.31 (95% CrI: 1.24, 1.39). Neighborhoods that were HCSAs had an infection rate 1.15 times greater (95% CrI: 1.08, 1.24) than non-HCSA neighborhoods. These results and their summary statistics were unchanged in the sensitivity analyses.

**Table 2:** Results from Bayesian spatial regression model of CA-Sa SSTI risk in California neighborhoods (*n* = 541)

	IRR	95% CrI
Variable		
Percent of working age adults	0.92	(0.87, 0.97)
Percent identifying as race/ethnicity other than NH-white	0.95	(0.93, 0.97)
Rural MSSA	0.92	(0.85, 1.01)
Percent Adult Poverty	1.31	(1.24, 1.39)
Healthcare shortage area	1.15	(1.08, 1.24)

Posterior estimates (infection risk ratio (IRR), 95% credible interval (CrI)) of the covariate coefficients; working age is 18–64 years; NH is non-Hispanic

## Discussion

In this study, we investigated the presence of geographic disparities in CA-Sa SSTI rates in California neighborhoods from a statewide, longitudinal database of ED electronic health records. To the best of our knowledge, this is one of the first studies to generate a risk map for CA-Sa SSTIs across California EDs. Our results suggest significant spatial variation in infection rates across California neighborhoods. Clusters of high IRRs were identified among neighborhoods in more rural, northern parts of California, a major departure from previous studies documenting high CA-Sa infection rates mainly in urban areas [5–7, 12, 15, 25, 45, 59–61].

We found weak associations between population structure and infection rates. While other geographies or populations may be more influenced by population structure, our results indicate that other factors likely explain community-level rates of CA-Sa SSTIs in California. At the neighborhood level, studies have documented persistent links between area-level poverty rates and a higher burden of CA-Sa infections [61–63]. HCSAs are defined by reduced primary health care access, an important component of infectious disease dynamics [52]. Disease patterns identified in choropleth maps overlapped with the geographic distribution of the percentage of adult poverty and HCSAs, indicating possible associations. Results from our Bayesian regression with R-INLA support positive associations between area-level poverty rates, HCSAs, and CA-Sa SSTI rates. While exploring potential mechanisms explaining these associations was beyond the scope of this study, a previous analysis of this data identified environmental degradation as a possible mechanism explaining the association between area-level poverty and CA-Sa infection [64]. Further, disparities in healthcare access may result in observed geographic disparities through individual-level factors such as health insurance coverage and type or area-level factors such as spatial accessibility [65].

Strengths of this study include using MSSAs to define neighborhoods maximizes homogeneity in the social and structural environment. Census tracts composing MSSAs have similar racial/ethnic compositions, poverty rates, age distribution, insurance status, and population densities. As California’s designated rational service areas for healthcare services, MSSAs are more amendable and translatable boundaries for health and policy interventions. Using HCAI ED data provided a comprehensive view of all CA-Sa SSTIs in California EDs. Finally, to the best of our knowledge, this study provides a more recent, comprehensive, and updated overview of CA-Sa SSTI rates than in the current published literature.

This study has several limitations, including associative modeling of areal data, which is subject to ecological bias and modifiable areal unit problems. While ecological biases do not affect cluster detection or prediction of CA-Sa SSTI incidence rates, findings from our associative model are subject to ecological fallacy [66]. Further, we do not have laboratory confirmation of CA-Sa in the cases identified. Instead, we are using ICD codes for SSTIs most likely caused by CA-Sa, which are limited in the amount and quality of information collected at admission and also subject to potential coder errors [67]. Our results may reflect the geographic distribution of SSTIs more broadly than CA-Sa SSTIs. Future studies should conduct similar analyses among culture-confirmed cases. However, in the United States, only about 38% of SSTI patients are cultured [68], so a thorough understanding of the epidemiology for CA-Sa SSTIs will require synthesizing information from both study designs.

Our hotspot analyses identified areas of high infection clustering in the more rural, northern parts of the state and a few southeastern neighborhoods. However, in our model-based IRR estimations, the southeastern areas had only a slightly higher IRR than the surrounding neighborhoods. The clustering of these areas may have been influenced by the size and location of the polygons, low population densities, having few geographic neighbors, and possibly by edge effects as they reside along the California border [69]. Additionally, using MSSAs as our neighborhood definition may limit direct comparability to other CA-Sa study geographies. However, MSSAs are comprised of census tracts and do not cross county lines and can be compared qualitatively to other CA-Sa studies using census tracts or counties as the geographic analysis unit. Finally, our research only evaluates individuals seeking healthcare in the ED, which may only capture severe or advanced cases or be more reflective of populations who utilize ED care frequently, such as those with low income, certain racial/ethnic minorities, and individuals without access to primary care [36]. Future research should use datasets from other outpatient settings to examine how CA-Sa infections are distributed across California and compare findings.

## Conclusion

Our study is consistent with previous findings showing geographic differences in CA-Sa SSTI rates [12, 13, 17]. It builds on this knowledge by evaluating the geographic distribution of infections in a larger, more heterogeneous geographic area and using a unique neighborhood definition, California MSSA. Evidence suggests that geographic areas at increased risk for CA-Sa SSTIs may have shifted from cities and urban areas at the height of the CA-MRSA pandemic to more rural locations. Specifically, several neighborhoods in northern California had a disproportionately high IRR, which was associated with but only partially explained by area-level poverty rates or healthcare access.

CA-Sa is rarely life-threatening. However, the increasing resistance of *S. aureus* bacteria to antibiotics is part of a larger issue that greatly concerns healthcare and public health professionals [70]. Additionally, CA-Sa SSTIs can become chronic infections for populations with limited resources, significantly impacting quality of life [9–11]. Our unique neighborhood definition allowed us to quantify disease incidence in these at-risk populations as California HCAI uses MSSAs to identify areas of unmet healthcare needs and inform the allocation of public health funds [32]. The parts of California with high infection rates in this study also have a high percentage of the population living below the FPL, an association well documented in the literature and indicative of a potential poverty trap [62, 71, 72].

While our analysis was not meant to explore causative factors, the associations identified between healthcare shortage areas, high poverty rates, and infection suggest these neighborhoods could benefit from increased resources. Prioritizing issues like improved healthcare access, and more thorough investigations into the influence of modifiable determinants that could be driving these geographic disparities – like sanitation and hygiene infrastructure, risks from injection drug use, and environmental degradation – are needed to identify and mitigate potential inequities in infection burden [63, 64]. Future studies should also investigate the CA-Sa burden in other geographically varied populations to identify whether this shift in epidemiology holds across other states and populations.

### Supplementary Information

**Additional file 1: Table A1.** List of California Medical Service Study Areas (MSSA) that have high-high rates of CA-MRSA clustering (HH), low-low rates of CA-MRSA clustering (LL), and low-high rates of CA-MRSA clustering (LH), 2016-2019. **Table A2.** Ten California MSSAs with the highest and lowest risk ratio for CA-MRSA between 2016-2019*. ***A3.** Analysis code - R-Markdown html file.

## Data Availability

The data supporting this study’s findings are available from California’s Department of Healthcare Access and Information. However, restrictions apply to the availability of these data, which were used under license for the current study due to their containing protected health information, and so are not publicly available. Data are, however, available from the authors upon reasonable request and with permission from the California Department of Healthcare Access and Information. The authors have made the accompanying R code available.

## Abbreviations

CA-MRSA: Community-acquired Methicillin-resistant *Staphylococcus aureus*
CA-Sa: Community-acquired *Staphylococcus aureus*
CA-MSSA: Community-acquired Methicillin-susceptible *Staphylococcus aureus*
SSTI: Skin and soft-tissue infection
SES: Socioeconomic status
MSSA: Medical service study area
HCAI: California’s Department of Healthcare Access and Information
ED: Emergency department
ICD: International Classification of Disease
SIR: Standardized Infection Ratio
IRR: Infection risk ratio
FPL: Federal poverty level
HCSA: Healthcare shortage area
CrI: Credible interval
