# Supplementary material for: Community-acquired Staphylococcus aureus skin and soft tissue infection risk assessment using hotspot analysis and risk maps: the case of California emergency departments
Source: BMC Public Health. 2024 Jan 9;24:123. doi: 10.1186/s12889-023-17336-6 (PMC10775506; doi:10.1186/s12889-023-17336-6)
Supplement: Supplementary file 1 — Additional file 1: Table A1. List of California Medical Service Study Areas (MSSA) that have high-high rates of CA-MRSA clustering (HH), low-low rates of CA-MRSA clustering (LL), and low-high rates of CA-MRSA clustering (LH), 2016-2019. Table A2. Ten California MSSAs with the highest and lowest risk ratio for CA-MRSA between 2016-2019. A3. Analysis code - R-Markdown html file. [file 12889_2023_17336_MOESM1_ESM.zip › CA-Sa Spatial Analysis Appendices.docx]

## Supplementary Material

**Table A1: List of California Medical Service Study Areas (MSSA) that have high-high rates of CA-MRSA clustering (HH), low-low rates of CA-MRSA clustering (LL), and low-high rates of CA-MRSA clustering (LH), 2016-2019.**

| **MSSA** | **Cluster Type** |
| --- | --- |
| Adin/Lookout/Newell | HH |
| Alabama Hills/Cartago/Darwin/Keeler/Lone Pine/Lone Pine Paiute Shoshone Tribe/Olancha/Pearsonville | HH |
| Anderson/Cottonwood/French Gulch/Happy Valley/Igo/Ono/Platina/Shasta | HH |
| Arcata/Eureka | HH |
| Artois/Elk Creek/Glenn/Grindstone Indian Rancheria/Willows | HH |
| Bailey Creek/Canyondam/Chester/Eastshore/Foxwood/Hamilton Branch/Lake Almanor Peninsula/ Lake Almanor Peninsula/Lake Almanor West/Prattville/Warner Valley | HH |
| Belden/Caribou/Crescent Mills/Genessee/Greenville/Indian Falls/North Arm/Storrie/Taylorsville/Tobin/ Twain | HH |
| Bella Vista/City of Shasta Lake | HH |
| Big Bend/Montgomery Creek/Oak Run/Round Mountain | HH |
| Blairsden/Clio/C-Road/Gold Mountain/Greagle/Johnsville/Mohawk Meadows/Mohawk Vista/ Plumas Eureka/Plumas Pines/Sierra Springs/Valley Ranch/White Hawk | HH |
| Bluelake/McKinleyville/Orick/Trinidad | HH |
| Boonville/Navarro/Philo/Yorkville | HH |
| Boron/California City/Desert Lake/Mojave/North Edwards/Rosamond | HH |
| Brooktrails/Pine Mountain/Willits | HH |
| Bucks Lake/Cromberg/East Quincy/Greenhorn/Keddie/Laporte/Little Grass Valley/Meadow Valley/ Quincy/Sloat/Spring Garden | HH |
| Butte Meadows/Cohasset/Forest Ranch | HH |
| Carrick/Edgewood/Mount Shasta/Weed | HH |
| Castella/Lakehead/O’Brien | HH |
| Ceres/Modesto South Central | HH |
| Clearlake/Clearlake Oaks | HH |
| Cobb/Hidden Valley/Middletown | HH |
| Covelo/Dos Rios | HH |
| Crescent City/Gasquet/Klamath/Smith River | HH |
| Dairyville/Manton/Mineral/Paynes Creek | HH |
| Douglas City/Lewiston/Trinity Center/Weaverville | HH |
| Dunsmuir | HH |
| Elk/Little River/Mendocino | HH |
| Etna/Fort Jones/Greenview | HH |
| Ferndale/Fortuna/Rio Dell/Scotia | HH |
| Forest Glen/Hayfork/Hyampom/Peanut | HH |
| Fort Bragg/Westport | HH |
| Garberville/Redway | HH |
| Gerber/Los Flores/Proberta/Red Bluff | HH |
| Greenhaven/Land Park/Midtown/Pocket/Riverside/Sutterville | HH |
| Happy Camp | HH |
| Hoopa/Willow Creek | HH |
| Hopland | HH |
| Inyokern/Ridgecrest | HH |
| Junction City/Salyer | HH |
| Kelseyville/Lakeport | HH |
| Kettenpom/Mad River/Ruth/Xenia | HH |
| Laytonville/Leggett/Piercy | HH |
| Loduga/Maxwell/Princeton/Stonyford | HH |
| Lower Lake | HH |
| Lucerne/Nice/Upper Lake | HH |
| Manton/Millville/Shingletown/Viola | HH |
| McCloud/Tennant | HH |
| Modesto East | HH |
| Palo Cedro | HH |
| Paskenta/Rancho Tehama | HH |
| Plymouth | HH |
| Potter Valley | HH |
| Redding | HH |
| Redwood Valley | HH |
| Talmage | HH |
| Ukiah | HH |
| Alviso/Palo Alto Bayshore/Santa Clara Northwest/Sunnyvale North | LL |
| Anaheim Hills/City of Orange East/Villa Park | LL |
| Cambrian Village West/Campbell South/Los Gatos/Monte Sereno/San Tomas | LL |
| Carmel Valley/Del Dios/Escondido South/Fairbanks Ranch/Poway Southeast/Rancho Bernardo Southeast/Rancho Penasquitos | LL |
| Clairemont/Fiesta Shores/Linda Vista/Mission Beach/Sorrento/University City | LL |
| Coto de Caza/Las Flores/Mission Viejo Northwest/Rancho Santa Margarita/Trabuco Canyon | LL |
| Cupertino/Rancho Rinconada/San Jose West/Saratoga | LL |
| Dana Point/San Clemente/San Juan Capistrano | LL |
| Escondido East/Hidden Meadows/Poway North | LL |
| Foothill Ranch/IrvineNortheast/Lake Forest West/Portola Hills/Tustin East | LL |
| Fremont South/Mission San Jose/Newark South/Warm Springs | LL |
| Laguna Beach/Laguna Woods | LL |
| Lake Forest East/Mission Viejo Central and South | LL |
| Los Altos/Los Altos Hills/Palo Alto Central/Stanford | LL |
| Pasadena South/San Marino/South Pasadena | LL |
| Poway Central/Poway Grove/Rancho Bernardo Southwest/Sabre Springs | LL |
| Santa Clara South and West | LL |
| Sunnyvale South | LL |
| Baker/Harvard/Newberry Springs | LH |
| Chinese Camp | LH |
| Chiriaco Summit/Desert Center/Eagle Mountain | LH |
| Esparto/Rumsey | LH |
| Surprise Valley | LH |
| Westwood | LH |
| Williams | LH |

**Table A2**: **Ten California MSSAs with the highest and lowest risk ratio for CA-MRSA between 2016-2019**

|  | Risk Ratio | 95% Credible Interval |
| --- | --- | --- |
| Medical Service Study Area |  |  |
| Clearlake/Clearlake Oaks | 5.93 | 5.69, 6.17 |
| Lucerne/Nice/Upper Lake | 4.73 | 4.43, 5.04 |
| Dunsmuir | 4.36 | 3.75, 5.04 |
| Alta Sierra/Bodfish/Glenville/Kernville/Lake Isabella/Weldon/Wofford Heights | 3.85 | 3.63, 4.08 |
| Crescent City/Gasquet/Klamath/Smith River | 3.73 | 3.57, 3.91 |
| Bailey Creek/Canyondam/Chester/East Shore/Foxwood/Hamilton Branch/Lake Almanor Peninsula/Lake Almanor West/Prattville/Warner Valley | 3.65 | 3.23, 4.10 |
| Garberville/Redway | 3.63 | 3.34, 3.94 |
| Brooktrails/Pine Mountain/Willits | 3.58 | 3.36, 3.82 |
| Ukiah | 3.55 | 3.40, 3.71 |
| Big River/Needles | 3.52 | 3.20, 3.85 |
| Mammoth Lakes | 0.14 | 0.10, 0.20 |
| Carmel Valley/Del Dios/Escondido South/Fairbanks Ranch/Poway Southeast/Rancho Bernardo Southeast/Rancho Penasquitos | 0.31 | 0.30, 0.33 |
| Foothill Ranch/Irvine Northeast/Lake Forest West/Portola Hills/Tustin East | 0.31 | 0.30, 0.33 |
| Cupertino/Rancho Rinconada/San Jose West/Saratoga | 0.33 | 0.31, 0.36 |
| Mira Mesa/Scripps Miramar Ranch | 0.35 | 0.33, 0.37 |
| Sunnyvale South | 0.38 | 0.36, 0.41 |
| Irvine Central/Tustin East | 0.38 | 0.36, 0.41 |
| Alamo/Danville/Diablo/San Ramon | 0.40 | 0.38, 0.43 |
| Coto de Caza/Las Flores/Mission Viejo Northwest/Rancho Santa Margarita/Trabuco Canyon | 0.41 | 0.39, 0.43 |
| Golden Gate Park/Parkside/Sunset/West Portal | 0.41 | 0.38, 0.44 |

Controlling for population structure (percent working age, percent identifying as race/ethnicity other than non-Hispanic white, and rurality), the percent of adults over the age of 18 years living below the federal poverty level, and whether the MSSA is a healthcare shortage area.

**A3. Analysis code - R-Markdown html file**
