# Supplementary material for: Community-acquired Staphylococcus aureus skin and soft tissue infection risk assessment using hotspot analysis and risk maps: the case of California emergency departments
Source: BMC Public Health. 2024 Jan 9;24:123. doi: 10.1186/s12889-023-17336-6 (PMC10775506; doi:10.1186/s12889-023-17336-6)
Supplement: Supplementary file 1 — Additional file 1: Table A1. List of California Medical Service Study Areas (MSSA) that have high-high rates of CA-MRSA clustering (HH), low-low rates of CA-MRSA clustering (LL), and low-high rates of CA-MRSA clustering (LH), 2016-2019. Table A2. Ten California MSSAs with the highest and lowest risk ratio for CA-MRSA between 2016-2019. A3. Analysis code - R-Markdown html file. [file 12889_2023_17336_MOESM1_ESM.zip › BLMB_CASa_Spatial Analysis.html]

CA-MRSA Hotspot Analysis & Risk Maps


# CA-MRSA Hotspot Analysis & Risk Maps

#### BL Morgan Bustamante

#### 2023-07-28

# Community-Acquired Methicillin-resistant *Staphylococcus aureus* disease risk assessment using hotspot analysis and risk maps: the case of California

# 1. Data Wrangling & Exploration

```
library(sf)
```

```
## Linking to GEOS 3.10.2, GDAL 3.4.2, PROJ 8.2.1; sf_use_s2() is TRUE
```

```
library(dplyr)
```

```
## 
## Attaching package: 'dplyr'
```

```
## The following objects are masked from 'package:stats':
## 
##     filter, lag
```

```
## The following objects are masked from 'package:base':
## 
##     intersect, setdiff, setequal, union
```

```
library(tidyverse)
```

```
## ── Attaching packages
## ───────────────────────────────────────
## tidyverse 1.3.2 ──
```

```
## ✔ ggplot2 3.4.0     ✔ purrr   1.0.1
## ✔ tibble  3.1.8     ✔ stringr 1.5.0
## ✔ tidyr   1.3.0     ✔ forcats 0.5.2
## ✔ readr   2.1.3     
## ── Conflicts ────────────────────────────────────────── tidyverse_conflicts() ──
## ✖ dplyr::filter() masks stats::filter()
## ✖ dplyr::lag()    masks stats::lag()
```

```
library(tmap)
library(tmaptools)
library(RColorBrewer)
library(spdep)
```

```
## Loading required package: sp
## Loading required package: spData
## To access larger datasets in this package, install the spDataLarge
## package with: `install.packages('spDataLarge',
## repos='https://nowosad.github.io/drat/', type='source')`
```

```
library(rsconnect)

setwd("/Users/brittanymorgan/Desktop/Data")
load(file = "/Users/brittanymorgan/Desktop/Data/mssa.df")
load(file = "/Users/brittanymorgan/Desktop/Data/df.age")
load(file = "/Users/brittanymorgan/Desktop/Data/df.zipcode")
load(file = "/Users/brittanymorgan/Desktop/Data/ssti.tract")
mssa.census <- st_read("/Users/brittanymorgan/Desktop/Data/MSSA_Detail/MSSA_Detail.shp") %>%
            select(census = CENSUS_KEY, tpop = POP_CIV, geometry)
```

```
## Reading layer `MSSA_Detail' from data source 
##   `/Users/brittanymorgan/Desktop/Data/MSSA_Detail/MSSA_Detail.shp' 
##   using driver `ESRI Shapefile'
## Simple feature collection with 8036 features and 62 fields
## Geometry type: MULTIPOLYGON
## Dimension:     XY
## Bounding box:  xmin: -124.4097 ymin: 32.53416 xmax: -114.1312 ymax: 42.00952
## Geodetic CRS:  WGS 84
```

```
zipfile <- st_read("/Users/brittanymorgan/Desktop/Data/zcta/ZCTA2010.shp") %>%
            select(ZCTA, TOT_POP, geometry)
```

```
## Reading layer `ZCTA2010' from data source 
##   `/Users/brittanymorgan/Desktop/Data/zcta/ZCTA2010.shp' using driver `ESRI Shapefile'
## Simple feature collection with 1769 features and 85 fields
## Geometry type: MULTIPOLYGON
## Dimension:     XY
## Bounding box:  xmin: -373976.1 ymin: -604516.1 xmax: 540037.5 ymax: 450043.5
## Projected CRS: NAD83 / California Albers
```

```
# combine age-stratified cases by zip code
df.zip2 <- df.zip %>%
  ungroup() %>%
  select(zcta, cases) %>%
  group_by(zcta) %>%
  mutate(ncases = sum(cases)) %>%
  select(zcta, ncases) %>%
  unique()

# attach zcta shapefile
zip.df <- left_join(zipfile, df.zip2, by = c("ZCTA" = "zcta")) %>%
  mutate(rt = (ncases/TOT_POP)*10000)

# suppress zctas where estimated total population is less than 20 individuals
zip.supp <- zip.df %>%
  filter(TOT_POP < 20)
zip.map <- zip.df %>%
  filter(TOT_POP > 20)

# calculate case rate for census tracts
col_list <- c("2016", "2017", "2018", "2019")
ssti.tract <- ssti.tract %>%
  mutate(cases = sum(c_across(all_of(col_list))))
tract.map <- left_join(mssa.census, ssti.tract, by = c("census" = "tract")) %>%
  mutate(rt = (cases/tpop)*10000)

# suppress census tracts where estimated total population is less than 20 individuals
census.supp <- tract.map %>%
  filter(tpop < 20)
census.map <- tract.map %>%
  filter(tpop > 20)


mssa.df <- st_transform(mssa.df, crs = "+proj=utm +zone=10 +datum=NAD83 +ellps=GRS80")
df.age <- st_transform(df.age, crs = "+proj=utm +zone=10 +datum=NAD83 +ellps=GRS80")
zip.df <- st_transform(zip.df, crs = "+proj=utm +zone=10 +datum=NAD83 +ellps=GRS80")

# calculate percent of adults over the age 18 living below FPL & yearly rates
mssa.df$yr16 <- mssa.df$`2016`
mssa.df$yr17 <- mssa.df$`2017`
mssa.df$yr18 <- mssa.df$`2018`
mssa.df$yr19 <- mssa.df$`2019`

mssa.df <- mssa.df %>%
  mutate(adultpov = 100*(pov18.34 + pov35.64 + pov65)/povdenom,
         rt.16 = 10000*(yr16/tpop),
         rt.17 = 10000*(yr17/tpop),
         rt.18 = 10000*(yr18/tpop),
         rt.19 = 10000*(yr19/tpop))

# rate per year
rt <- as.data.frame(mssa.df) %>%
  select(tpop, yr16, yr17, yr18, yr19) %>%
  dplyr::summarize(across(everything(), sum)) %>%
  mutate(rt16 = 10000*(yr16/tpop), rt17 = 10000*(yr17/tpop), 
         rt18 = 10000*(yr18/tpop), rt19 = 10000*(yr19/tpop))
```

First we will evaluate the spatial variation in CA-MRSA infections
per 10,000 individuals by using

1. ZCTA as the geographic analysis unit

```
# ZCTA map
zip <- tm_shape(zip.map) +
  tm_polygons(col = "rt", style = "fisher",
              palette = get_brewer_pal(palette = "Reds", n = 5),
              boder.col = "black",
              border.alpha = 0.1,
              legend.show = FALSE) +
tm_shape(zip.supp) +
  tm_polygons(col = "black") +
tm_add_legend("fill",
              col = c("#FEE4D8", "#FCB195", "#FB795A", "#EF3C2D", "#BB1419", 
                      "#808080", "#000000"),
              labels = c("4 to 457", "457 to 2,687", "2,687 to 12,255", "12,255 to 20,382",
                         "20,381 to 24,632", "Missing", "Suppressed"),
              title = "Infections per 10k",
              border.lwd = 0)
```

```
# tmap_save(zip, "figure1a.png", width = 800, dpi = 300)
zip
```

2. the randomly assigned census tract as the geographic analysis
   unit

```
# census map
census.fix <- st_make_valid(census.map)
census.supp.fix <- st_make_valid(census.supp)

census <- tm_shape(census.fix) +
  tm_polygons(c("rt"), style = "fisher",
              palette = get_brewer_pal(palette = "Reds", n = 5),
              boder.col = "black",
              border.alpha = 0.1,
              legend.show = FALSE) +
tm_shape(census.supp.fix) +
  tm_polygons(col = "black") +
tm_add_legend("fill",
              col = c("#FEE4D8", "#FCB195", "#FB795A", "#EF3C2D", "#BB1419", 
                      "#808080", "#000000"),
              labels = c("5 to 291", "291 to 796", "796 to 4,373", "4,373 to 20,275",
                         "20,275 to 32,414", "Missing", "Suppressed"),
              title = "Infections per 10k",
              border.lwd = 0)
```

```
# tmap_save(census, "figure1b.png", width = 800, dpi = 300)
census
```

3. Crosswalked MSSA as the geographic analysis unit

```
# MSSA map
mssa.map <- tm_shape(mssa.df) +
  tm_polygons(c("sstirt"), style = "jenks",
              palette = get_brewer_pal(palette = "Reds", n = 5),
              boder.col = "black",
              border.alpha = 0.1,
              legend.show = FALSE) +
tm_add_legend("fill",
              col = c("#FEE4D8", "#FCB195", "#FB795A", "#EF3C2D", "#BB1419"),
              labels = c("22 to 188", "188 to 300", "300 to 470", "470 to 733",
                         "733 to 1,175"),
              title = "Infections per 10k",
              border.lwd = 0)
```

```
# tmap_save(mssa.map, "figure1c.png", width = 800, dpi = 300)
mssa.map
```

The first part of this chapter uses disease mapping methods to
estimate CA-MRSA risk in California in years 2016-2019. This analysis is
conducted at the Medical Service Study Area level, the OSHPD defined
rational health service area. The dataframe contains the age-stratified
population and the age-stratified number of CA-MRSA cases. Population
data was obtained from the 2019 ACS 5-year estimates, the CA-MRSA cases
were obtained from the California Department of Health Care Access and
Information.

## 1.1 Expected Cases

We will calculate the number of expected cases in each California
MSSA using the expected() function in the SpatialEpi package. There are
three age groups for each MSSA, so the number of strata is set to
three.

```
library(SpatialEpi)

# sort data as MSSA then age
df.age <- df.age[order(df.age$mssa, df.age$agecat), ]

E <- expected(population = df.age$population, cases = df.age$cases, n.strata = 3)

# add age-stratified expected cases to the mssa dataframe
mssa.df$E <- E

head(mssa.df)
```

```
## Simple feature collection with 6 features and 47 fields
## Geometry type: GEOMETRY
## Dimension:     XY
## Bounding box:  xmin: 627870.6 ymin: 3738349 xmax: 1108565 ymax: 4650972
## CRS:           +proj=utm +zone=10 +datum=NAD83 +ellps=GRS80
## # A tibble: 6 × 48
## # Rowwise: 
##   mssa        tpop popdens pctnhw pctnhb pctnh…¹ pctnhai pcthisp pctim…² pctnohs
##   <chr>      <dbl>   <dbl>  <dbl>  <dbl>   <dbl>   <dbl>   <dbl>   <dbl>   <dbl>
## 1 Aberdeen…   2284 8.70e-1   58.9  0.832    1.14  19.4      14.6  0.0876   27.3 
## 2 Acton/Ra…  11945 5.32e+1   72.5  0.854    2.90   0.167    21.8  1.41      2.61
## 3 Adelanto…  64726 2.08e+2   20.1 17.0      3.54   0.224    56.0  0.780     9.18
## 4 Adin/Loo…   1650 1.73e+0   69.1  0        0      3.15     25.7  0       NaN   
## 5 Agoura H… 119647 1.23e+3   71.0  2.63    10.0    0.269    11.9  1.44      4.60
## 6 Agua Cal…  30281 2.98e+2   65.7  2.30     4.59   0.624    25.1  0.974     5.87
## # … with 38 more variables: pctcrowd <dbl>, pctpov <dbl>, tot18.34 <dbl>,
## #   tot35.64 <dbl>, tot65 <dbl>, pov18.34 <dbl>, pov35.64 <dbl>, pov65 <dbl>,
## #   povdenom <dbl>, avgscore <dbl>, ozone <dbl>, pm25 <dbl>, diesel <dbl>,
## #   pesticide <dbl>, water <dbl>, groundwater <dbl>, hazwaste <dbl>,
## #   waterbodies <dbl>, solwaste <dbl>, pollburdenavg <dbl>, rural <chr>,
## #   `2016` <dbl>, `2017` <dbl>, `2018` <dbl>, `2019` <dbl>,
## #   geometry <GEOMETRY [m]>, cases <dbl>, sstirt <dbl>, yr16 <dbl>, …
```

## 1.2 Age-Standardized Infection Ratios

For each MSSA, the SIR is defined as the ratio of observed counts to
the expected counts.

```
mssa.df$SIR <- mssa.df$cases/mssa.df$E
```

## 1.3 Exploratory Analysis

```
# map cases 2016
tm_shape(mssa.df) +
  tm_polygons(c("rt.16"), breaks = c(0, 50, 100, 150, 200, 250, Inf),
              palette = "Reds",
              boder.col = "black",
              border.alpha = 0.1,
              title = "Infections per 10k Residents") +
  tm_layout(main.title = "CA-MRSA Infections in California, 2016",
            main.title.size = 0.95, frame = FALSE,
            legend.outside = TRUE, legend.outside.position = "right")
```

```
# map cases 2017
tm_shape(mssa.df) +
  tm_polygons(c("rt.17"), breaks = c(0, 50, 100, 150, 200, 250, Inf),
              palette = "Reds",
              boder.col = "black",
              border.alpha = 0.1,
              title = "Rates per 10k Residents") +
  tm_layout(main.title = "CA-MRSA Infections in California, 2017",
            main.title.size = 0.95, frame = FALSE,
            legend.outside = TRUE, legend.outside.position = "right")
```

```
# map cases 2018
tm_shape(mssa.df) +
  tm_polygons(c("rt.18"), breaks = c(0, 50, 100, 150, 200, 250, Inf),
              palette = "Reds",
              boder.col = "black",
              border.alpha = 0.1,
              title = "Rates per 10k Residents") +
  tm_layout(main.title = "CA-MRSA Infections in California, 2018",
            main.title.size = 0.95, frame = FALSE,
            legend.outside = TRUE, legend.outside.position = "right")
```

```
# map cases 2019
tm_shape(mssa.df) +
  tm_polygons(c("rt.19"), breaks = c(0, 50, 100, 150, 200, 250, Inf),
              palette = "Reds",
              boder.col = "black",
              border.alpha = 0.1,
              title = "Rates per 10k Residents") +
  tm_layout(main.title = "CA-MRSA Infections in California, 2019",
            main.title.size = 0.95, frame = FALSE,
            legend.outside = TRUE, legend.outside.position = "right")
```

```
# map infection rates combined 2016-2019
tm_shape(mssa.df) +
  tm_polygons(c("sstirt"), style = "pretty",
              palette = "Reds",
              boder.col = "black",
              border.alpha = 0.1,
              title = "Rates per 100k Residents") +
  tm_layout(main.title = "CA-MRSA Infections in California,
            2016-2019",
            main.title.size = 0.95, frame = FALSE,
            legend.outside = TRUE, legend.outside.position = "right")
```

```
# graph temporal trends
year <- c("2016", "2017", "2018", "2019")
casert <- c(rt$rt16, rt$rt17, rt$rt18, rt$rt19)
graph.rt <- data.frame(year, casert)

ggplot(graph.rt, aes(x = year, y = casert, group = 1)) +
  geom_line(linetype = "dashed") +
  geom_point() +
  ylim(0, 800) +
  labs(x = "Year", y = "Rate per 100K Residents",
       title = "CA-MRSA Infections in California Emergency Departments, 2016-2019") +
  theme_minimal() +
  theme(legend.position = "none")
```

```
summary(mssa.df$pctpov)
```

```
##    Min. 1st Qu.  Median    Mean 3rd Qu.    Max. 
##   2.281   8.554  12.661  14.259  18.923  44.366
```

```
# map percent of adults living below the federal poverty limit
pov <- tm_shape(mssa.df) +
  tm_polygons(c("pctpov"), style = "jenks",
              palette = "Reds",
              boder.col = "black",
              border.alpha = 0.1,
              title = "Percent",
              legend.format = list(fun = function(x) paste0(formatC(x, digits = 0, 
                                                                    format = "f"), "%")))

# tmap_save(pov, "figure2e.png", width = 800, dpi = 300)
pov
```

```
# graph mean infection rate by poverty
mssa.df <- mssa.df %>%
  mutate(povcat = cut(pctpov, breaks = c(-Inf, 5, 10, 15, Inf), 
                                labels = c("less than 5%",
                                           "5 to 9.99%",
                                           "10 to 14.99%",
                                           "15% and over")))

pov.cat <- mssa.df %>%
  group_by(povcat) %>%
  dplyr::summarize(meanrt = mean(sstirt), sd = sd(sstirt))

ggplot(pov.cat, aes(x = povcat, y = meanrt)) +
  geom_bar(position = position_dodge(), stat = "identity", fill = "lightblue",
           color = "black") +
  geom_errorbar(aes(ymin = meanrt-sd, ymax = meanrt+sd), width = 0.2) +
  labs(x = "Percent of Adults Below FPL", y = "Mean (SD) CA-MRSA Rate",
       title = "Mean number of CA-MRSA infections per 10k residents by percent of adults 
       living below the federal poverty level (FPL), 2016-2019") +
  theme_minimal()
```

```
hcsa <- read.csv(file = "/Users/brittanymorgan/Desktop/Data/Health_Professional_Shortage_Area_Primary_Care.csv")

# health care shortage areas
hcsa$hcsa <- 1
hcsa.flag <- hcsa %>%
  dplyr::select(mssa = MSSA_NAME, hcsa)

mssa.df <- left_join(mssa.df, hcsa.flag, by = c("mssa" = "mssa")) %>%
  mutate(hcsa = if_else(is.na(hcsa), 0, hcsa))

# map
hcsa <- tm_shape(mssa.df) +
  tm_polygons(col = "hcsa", style = "cat", 
              labels = c("No", "Yes"),
              palette = "Reds", 
              border.col = "black",
              border.alpha = 0.1, 
              legend.hist = FALSE,
              title = "HCSA")

# tmap_save(hcsa, "figure2f.png", width = 800, dpi = 300)
hcsa
```

```
# variable for percent identifying as race/ethnicity other than non-Hispanic white
mssa.df$poc = (100-mssa.df$pctnhw)

# map
poc <- tm_shape(mssa.df) +
  tm_polygons(c("poc"), style = "jenks",
              palette = "Reds",
              boder.col = "black",
              border.alpha = 0.1,
              title = "Percent",
              legend.format = list(fun = function(x) paste0(formatC(x, digits = 0, 
                                                                    format = "f"), "%")))

# tmap_save(poc, "figure2c.png", width = 800, dpi = 300)
poc
```

```
# variable for percent working age
mssa.df$wrkage = (100*((mssa.df$tot18.34+mssa.df$tot35.64)/mssa.df$tpop))

# map
wrk <- tm_shape(mssa.df) +
  tm_polygons(c("wrkage"), style = "jenks",
              palette = "Reds",
              boder.col = "black",
              border.alpha = 0.1,
              title = "Percent",
              legend.format = list(fun = function(x) paste0(formatC(x, digits = 0, 
                                                                    format = "f"), "%")))

# tmap_save(wrk, "figure2b.png", width = 800, dpi = 300)
wrk
```

```
# rural area
mssa.df$ruralflag <- ifelse(mssa.df$rural == "Urban or Town", 0, 1)

# map
rural <- tm_shape(mssa.df) +
  tm_polygons(col = "ruralflag", style = "cat", 
              labels = c("No", "Yes"),
              palette = "Reds", 
              border.col = "black",
              border.alpha = 0.1, 
              title = "Rural",
              legend.hist = FALSE)

# tmap_save(rural, "figure2d.png", width = 800, dpi = 300)
rural
```

Statistics

```
table(mssa.df$hcsa)
```

```
## 
##   0   1 
## 315 227
```

```
table(mssa.df$rural)
```

```
## 
##      Frontier         Rural Urban or Town 
##            54           168           320
```

# 2. Mapping SIR

To visualize the observed and expected infection counts, the SIRs
will be mapped in an interactive choropleth map using leaflet.

```
library(leaflet)

# transform to EPSG code for for lat/long to map in leaflet
map <- st_transform(mssa.df, 4326)

l <- leaflet(map) %>% addTiles()

pal <- colorNumeric(palette = "YlOrRd", domain = map$SIR)

labels <- sprintf("<strong>%s</strong><br/>Observed: %s <br/>Expected: %s<br/> SIR: %s",
                  map$mssa, map$cases, round(map$E, 2), round(map$SIR, 2)) %>%
  lapply(htmltools::HTML)

l %>% addPolygons(color = "grey", weight = 1, fillColor = ~pal(SIR), fillOpacity = 0.5,
                  highlightOptions = highlightOptions(weight = 4),
                  label = labels,
                  labelOptions = labelOptions(style = list("font-weight" = "normal",
                                                           padding = "3px 8px"),
                                              textsize = "15px", direction = "auto")) %>%
  addLegend(pal = pal, values = ~SIR, opacity = 0.5, title = "SIR", position = "bottomright")
```

Areas with:

- SIR = 1, indicates observed cases are the same as expected
- SIR > 1, indicates observed cases are higher than expected
- SIR < 1, indicates observed cases are lower than expected

This map gives us a sense of the infection risk across California
MSSAs. However, age-standardized infection ratios may be misleading in
MSSAs with small populations and do not tell us whether there is
clustering or spatial autocorrelation. The next section of this chapter
will test for clustering and then use a model-based approach to evalaute
spatial autocorrelation and smooth over extreme values by borrowing
information from neighboring MSSAs.

```
# static map of SIR
sir <- tm_shape(map) +
  tm_polygons(c("SIR"), style = "jenks",
              palette = "Reds",
              boder.col = "black",
              border.alpha = 0.1,
              title = "SIR",
              legend.format = list(fun = function(x) paste0(formatC(x, digits = 2, 
                                                                    format = "f"))))

# tmap_save(sir, "figure2a.png", width = 800, height = 886.5881, dpi = 300)
sir
```

# 3. Evaluating Geographic Clusters

Based on the previous SIR map, it looks like infection rates cluster
geographically. In particular, there appears to be a high concentration
of infections in communities in the northern and maybe the eastern part
of the state.

To know if there are geographic clusters of infection, we’ll run a
hotspot analysis. To do so, we need to define:

1. Neighbor connectivity (who is your neighbor?)
2. Neighbor weights (how much does your neighbor matter?)

## 3.1 Neighbor Connectivity

*We’ll use Queen adjacency, defines neighbors as any neighbor
sharing a* *line segment (border) or a point (or vertex)*

```
df <- mssa.df %>%
  filter(mssa != "Avalon") # remove MSSA with zero neighbors (catalina island)

calb <- poly2nb(df, queen = T)

summary(calb)
```

```
## Neighbour list object:
## Number of regions: 541 
## Number of nonzero links: 3038 
## Percentage nonzero weights: 1.03799 
## Average number of links: 5.615527 
## Link number distribution:
## 
##   1   2   3   4   5   6   7   8   9  10  11  12  13 
##   2  10  52  84 128 113  80  35  18  11   3   4   1 
## 2 least connected regions:
## 78 241 with 1 link
## 1 most connected region:
## 21 with 13 links
```

## 3.2 Neighbor Weights

Next step is to assign weights to each neighbor relationship. This
determines *how much* each neighbor counts.

Weights for our Queen contiguity defined neighbor object:

```
calw <- nb2listw(calb, style = "W") # W = weights are row standardized
```

Visualize neighbor connections between MSSAs using the weight matrix
created from nb2listw().

```
centroids <- st_centroid(st_geometry(df))
plot(st_geometry(df), border = "grey60", reset = FALSE)
plot(calb, coords = centroids, add = T, col = "red")
```

## 3.3 Moran Scatterplot

Visually explore the apparent clustering in the SIR map more by
plotting standardized CA-MRSA rates on the x-axis and the standardized
average CA-MRSA rate of one’s neighbors (known as the spatial lag) on
the y-axis

Create Moran scatterplot using the Queen based spatial weights
matrix

```
moran.plot((df$sstirt), listw = calw, xlab = "Standardized CA-MRSA Rate",
                                      ylab = "Standardized Lagged CA-MRSA Rate",
           main = c("Moran Scatterplot for CA-MRSA Rate", "in California"))
```

Looks like a positive association - the higher your neighbors’
CA-MRSA rate, the higher your CA-MRSA rate

# 4. Global spatial autocorrelation

The SIR map and Moran scatterplot provide descriptive visualizations
of clustering (autocorrelation) in CA-MRSA rates. We also need a
quantitative and objective measure of the degree to which similar
features cluster. For this, I’ll use global measures of spatial
autocorrelation. A global index of spatial autocorrelation provides a
summary over the entire study area of the level of spatial similarity
observed among neighboring observations.

## 4.1 Moran’s I

*(the most popular test of spatial autocorrelation)*

Moran’s I for Queen Contiguity

```
moran.test(df$sstirt, calw, zero.policy = TRUE)
```

```
## 
##  Moran I test under randomisation
## 
## data:  df$sstirt  
## weights: calw    
## 
## Moran I statistic standard deviate = 19.059, p-value < 2.2e-16
## alternative hypothesis: greater
## sample estimates:
## Moran I statistic       Expectation          Variance 
##      0.4972920544     -0.0018518519      0.0006858755
```

Moran’s I is positive (0.50) and statistically significant (p-value
< 0.01). Moran’s I is a correlation (ranges from -1 to 1). General
rule of thumb is spatial autocorrelation > 0.3 and < -0.3 is
meaningful. Further, the p-value for the correlation is statistically
significant.

Can compute a p-value from a Monte Carlo simulation w/1,000
simulations.

```
moran.mc(df$sstirt, calw, zero.policy = TRUE, nsim = 1000)
```

```
## 
##  Monte-Carlo simulation of Moran I
## 
## data:  df$sstirt 
## weights: calw  
## number of simulations + 1: 1001 
## 
## statistic = 0.49729, observed rank = 1001, p-value = 0.000999
## alternative hypothesis: greater
```

Once again end up with a p-value < 0. 01. The Moran’s I statistic
of 0.497 represents the highest Moran’s I value out of the 1,000
simulations

# 5. Local spatial autocorrelation

Moran’s I & Geary’s c tell us whether clustering exists in the
area, it does not tell us *where* clusters are located. For that
we use Local Indicators of Spatial Autocorrelation (LISAs).

LISAs primary goal is to provide a local measure of similarity
between each unit’s value (CA-MRSA rates) and those of nearby cases. So,
rather than a single summary of spatial association like Moran’s I, we
get a measure for every unit in the study area. We then map each MSSAs
LISA value to give us insight into the location of areas with
comparatively high or low associations with neighboring values (i.e, hot
and cold spots)

## 5.1 Getis-Ord

*A popular local measure of spatial autocorrelation. Two versions,
Gi and Gi\*,* *will calculate each*

To plot object, need to coerce localg object into numeric and
sort.

```
df$localg <- as.numeric(localG(df$sstirt, calw))

df.sort <- df %>%
          arrange(localg)
```

localg variable contains the Z-scores for the Gi statistic.
Interpretation of the Z-score is same as normal: a large positive value
means a cluster of high CA-MRSA rates (hot spots) and a large negative
value means a cluster of low CA-MRSA rates (cold spots).

Then, we create a vector names *breaks* to designate the
cutoff points at the different significance levels (1% or 99%), (5% or
95%), and (10% or 90%) using the appropriate Z-scores. Set the minimum
and maximum Gi as the floor and ceiling.

```
breaks <- c(min(df.sort$localg), -1.96, -1.65, 1.65, 1.96, 2.58, 
            max(df.sort$localg))
```

Then we can map the clusters.

```
tm_shape(df.sort, unit = "mi") +
  tm_polygons(col = "localg", title = "Gi value", palette = "-RdBu",
              breaks = breaks) +
  tm_scale_bar(breaks = c(0, 10, 20), text.size = 1) +
  tm_compass(type = "4star", position = c("left", "bottom")) +
  tm_layout(frame = F, main.title = "California Medical Service Study Area
            CA-MRSA Clusters", legend.outside = T)
```

```
## Variable(s) "localg" contains positive and negative values, so midpoint is set to 0. Set midpoint = NA to show the full spectrum of the color palette.
```

Shows hot spots in the northwest part of the state and a bit in the
southeast. Cold spots along the southwest coast and parts of the bay
area?

*Gi only uses neighbors to calculate hot and cold spots. To
incorporate the location itself in the calculation, need to calculate
Gi\**

```
# nb object that includes the location itself as a neighbor
calb.self <- include.self(calb)

# create self-included spatial weights object
cal.w.self <- nb2listw(calb.self, style = "W", zero.policy = TRUE)

# rerun localG using self-included weights
df$localgstar <- as.numeric(localG(df$sstirt, cal.w.self))

# sort
df.sort <- df %>%
          arrange(localgstar)
```

```
# create breaks based on significance thresholds
breaks <- c(min(df.sort$localgstar), -2.58, -1.96, -1.65, 1.65, 1.96, 2.58,
            max(df.sort$localgstar))

tm_shape(df.sort, unit = "mi") +
  tm_polygons(col = "localgstar", title = "Gi* value", palette = "-RdBu",
              breaks = breaks,
              border.alpha = 0.1) +
  tm_scale_bar(breaks = c(0, 10, 20), text.size = 1) +
  tm_compass(type = "4star", position = c("left", "bottom")) +
  tm_layout(frame = F, main.title = "California Medical Service Study Area
                                    CA-MRSA infection clusters", 
            legend.outside = T)
```

```
## Variable(s) "localgstar" contains positive and negative values, so midpoint is set to 0. Set midpoint = NA to show the full spectrum of the color palette.
```

Create a categorical variable within df that designates tracts as
cold, hot, and not significant.

```
df.sort <- df.sort %>%
  mutate(gcluster = cut(localgstar, breaks = breaks, include.lowest = TRUE,
                        labels = c("Cold spot: 99% Confidence",
                                   "Cold spot: 95% Confidence",
                                   "Cold spot: 90% Confidence",
                                   "Not significant",
                                   "Hot spot: 90% Confidence",
                                   "Hot spot: 95% Confidence",
                                   "Hot spot: 99% Confidence")))

# map the variable
tm_shape(df.sort, unit = "mi") +
  tm_polygons(col = "gcluster", title = "", palette = "-RdBu",
              breaks = breaks,
              border.alpha = 0.1) +
  tm_scale_bar(breaks = c(0, 10, 20), text.size = 1) +
  tm_compass(type = "4star", position = c("left", "bottom")) +
  tm_layout(frame = F, main.title = "California Medical Service Study Areas
                                    CA-MRSA infection Clusters",
            legend.outside = T)
```

For simplification, can also eliminate significance levels and just
map hot and cold spots as MSSAs with Z-scores above and below +-1.96

```
breaks <- c(min(df.sort$localgstar), -1.96, 1.96, max(df.sort$localgstar))
df.sort <- mutate(df.sort, gcluster = cut(localgstar, breaks = breaks,
                                          include.lowest = TRUE, 
                                          labels = c("Cold spot", "None",
                                                     "Hot spot")))
```

```
mssa.ssti.map.g <- tm_shape(df.sort, unit = "mi") +
  tm_polygons(col = "gcluster", title = "Cluster Type", palette = "-RdBu",
              breaks = breaks,
              border.alpha = 0.1)
mssa.ssti.map.g
```

## 5.2 Local Moran’s I

*Another popular measure of local spatial autocorrelation*

```
# recalculate calw
calw <- nb2listw(calb, style = "W")

locali <- localmoran(df$sstirt, calw)
```

locali is a matrix with 5 columns: local statistic, expected local
statistic, variance, Z-score, and p-value. Save the local statistic and
Z-score into df

```
df$localmi <- as.numeric(locali[,1])
df$localz <- as.numeric(locali[,4])
```

Make statistically significant cluster identifiers for Z-scores
greater than or less than +-1.96

```
df.sort <- df %>%
          arrange(localz)

breaks <- c(min(df$localz), -1.96, 1.96, max(df$localz))

df.sort <- df.sort %>%
            mutate(mcluster = cut(localz, breaks = breaks, 
                                          include.lowest = TRUE, 
                                          labels = c("Negative Correlation", 
                                                     "Not Significant", 
                                                     "Positive Correlation")))
```

Map it

```
cal.ssti.map.mi <- tm_shape(df.sort, unit = "mi") +
  tm_polygons(col = "mcluster", title = "Cluster Type", palette = "-RdBu",
              breaks = breaks,
              border.alpha = 0.1) +
  tm_scale_bar(breaks = c(0, 10, 20), text.size = 1) +
  tm_compass(type = "4star", position = c("left", "bottom")) +
  tm_layout(frame = F, main.title = "California Medical Service Study Areas
                                    CA-MRSA infection clusters",
            legend.outside = T)
cal.ssti.map.mi
```

In the map above, positive values indicate similarity between
neighbors while negative values indicate dissimilarity. Meaning, high
values are similar values being clustered and low values are dissimilar
(high and low rates) values being clustered. Local Moran’s I captures
spatial outliers, it does not distinguish between High-High, High-Low,
and Low-High clusters

We can classify the significant locations as H-H and L-L spatial
clusters and H-L and L-H spatial outliers. *important to remember:
reference to H and L is relative to the mean of the* *variable,
should not be interpreted in an absolute sense*

First, we identify if clusters are clusters or outliers based on
p-value (significant and positive = cluster, significant and negative =
outlier) Then, we identify whether the value is high or low relative to
the mean

```
sigleve <- 0.05
meanVal <- mean(df$sstirt)

locali %<>% tibble::as_tibble() %>%
  magrittr::set_colnames(c("Ii","E.Ii","Var.Ii","Z.Ii","Pr(z > 0)")) %>%
  dplyr::mutate(coType = dplyr::case_when(
  `Pr(z > 0)` > 0.05 ~ "Insignificant",
  `Pr(z > 0)` <= 0.05 & Ii >= 0 & df$sstirt >= meanVal ~ "HH",
  `Pr(z > 0)` <= 0.05 & Ii >= 0 & df$sstirt < meanVal ~ "LL",
  `Pr(z > 0)` <= 0.05 & Ii < 0 & df$sstirt >= meanVal ~ "HL",
  `Pr(z > 0)` <= 0.05 & Ii < 0 & df$sstirt < meanVal ~ "LH"
))
```

Now add the CoType variable to the original df and map the
clusters

```
df$coType <- locali$coType %>%
  tidyr::replace_na("Insignificant")

cal.ssti.map.coType <- tm_shape(df, unit = "mi") +
  tm_fill("coType", palette = c("tomato3", "white", "lightblue", "blue"),
          labels = c("High-High", "Insignificant", "Low-High", "Low-Low"),
          title = "Cluster Type") +
  tm_borders(col = "grey40", alpha = 0.1) +
  tm_layout(frame = FALSE)

tmap_save(cal.ssti.map.coType, "figure3.png", width = 800, dpi = 300)
```

```
## Map saved to /Users/brittanymorgan/Library/Mobile Documents/com~apple~CloudDocs/Documents/School/UC Davis/Dissertation/MRSA/Chapter 1/Code/figure3.png
```

```
## Resolution: 800 by 886.5881 pixels
```

```
## Size: 2.666667 by 2.955294 inches (300 dpi)
```

```
cal.ssti.map.coType
```

There is clustering of high rates in the northwest part of the state
and the south eastern part. There are a few clusters of low rates along
the coast and some outliers of low CA-MRSA areas surrounded by high rate
areas. However, there are no areas with high CA-MRSA rates surrounded by
areas with low CA-MRSA rates.

# 6. Modeling

Model-based approaches to estimating disease risk can incorporate
spatial autocorrelation, covariates, and borrow information from
neighboring counties to improve local estimates, resulting in a
smoothing of extreme values based on small sample sizes (an issue of
small-area estimation for areas with small populations).

### 6.1 Model

We’ll build a Poisson model with:

- An intercept
- A coefficient for percent of adults over age 18 living below the
  federal poverty limit
- A structured spatial effect (using Besag model)
  - To allow areas near each other to be more similar
- An unstructured spatial effect
  - To allow for the possibility areas near each other might not be
    similar

### 6.2 Neighborhood matrix

```
library(INLA)
```

```
## Loading required package: Matrix
```

```
## 
## Attaching package: 'Matrix'
```

```
## The following objects are masked from 'package:tidyr':
## 
##     expand, pack, unpack
```

```
## Loading required package: foreach
```

```
## 
## Attaching package: 'foreach'
```

```
## The following objects are masked from 'package:purrr':
## 
##     accumulate, when
```

```
## Loading required package: parallel
```

```
## This is INLA_22.12.16 built 2022-12-23 13:43:44 UTC.
##  - See www.r-inla.org/contact-us for how to get help.
##  - To enable PARDISO sparse library; see inla.pardiso()
```

```
nb <- poly2nb(map)
nb
```

```
## Neighbour list object:
## Number of regions: 542 
## Number of nonzero links: 3038 
## Percentage nonzero weights: 1.034163 
## Average number of links: 5.605166 
## 1 region with no links:
## 64
```

Tells us 1 polygon has no neighbors

```
map.inla <- map %>%
  filter(mssa != "Avalon") # remove MSSA with zero neighbors

nb <- poly2nb(map.inla, queen = TRUE)
summary(nb)
```

```
## Neighbour list object:
## Number of regions: 541 
## Number of nonzero links: 3038 
## Percentage nonzero weights: 1.03799 
## Average number of links: 5.615527 
## Link number distribution:
## 
##   1   2   3   4   5   6   7   8   9  10  11  12  13 
##   2  10  52  84 128 113  80  35  18  11   3   4   1 
## 2 least connected regions:
## 78 241 with 1 link
## 1 most connected region:
## 21 with 13 links
```

Convert the nb list into a file with the representation of the
neighborhood matrix as required by INLA

```
nb2INLA("nb.adj", nb)
g <- inla.read.graph(filename = "nb.adj")
```

### 6.3 Inference using INLA

The two random effects model spatial residual variation and
unstructured noise. We create two vectors in the data that denote the
indices of these random effects, both are equal to 1….541 (total number
of mssas)

```
map.inla$re_u <- 1:541
map.inla$re_v <- 1:541
```

Specify the model formula, for u(i) random effect we’ll use the besag
model and for v(i) we’ll use iid. *control.predictor = list(compute =
TRUE) is to compute the posterior means of the predictors.*

```
# mutate percent variables so the scale is a 10% change rater than a 1% change
map.inla <- map.inla %>%
  mutate(pov = pctpov/10,
         wrk = wrkage/10,
         poc2 = poc/10)

# besag model
full.formula <- cases ~ pov + wrk + poc2 + ruralflag + hcsa +
                f(re_u, model = "besag", graph = g, scale.model = TRUE) + 
                f(re_v, model = "iid")
set.seed(101122)
res <- inla(full.formula, family = "poisson", data = map.inla, E = E, 
            control.compute = list(dic = TRUE, waic = TRUE, cpo = TRUE,
                                   return.marginals.predictor = TRUE),
            control.predictor = list(compute = TRUE))
```

### 6.4 Results

```
summary(res)
```

```
## 
## Call:
##    c("inla.core(formula = formula, family = family, contrasts = contrasts, 
##    ", " data = data, quantiles = quantiles, E = E, offset = offset, ", " 
##    scale = scale, weights = weights, Ntrials = Ntrials, strata = strata, 
##    ", " lp.scale = lp.scale, link.covariates = link.covariates, verbose = 
##    verbose, ", " lincomb = lincomb, selection = selection, control.compute 
##    = control.compute, ", " control.predictor = control.predictor, 
##    control.family = control.family, ", " control.inla = control.inla, 
##    control.fixed = control.fixed, ", " control.mode = control.mode, 
##    control.expert = control.expert, ", " control.hazard = control.hazard, 
##    control.lincomb = control.lincomb, ", " control.update = 
##    control.update, control.lp.scale = control.lp.scale, ", " 
##    control.pardiso = control.pardiso, only.hyperparam = only.hyperparam, 
##    ", " inla.call = inla.call, inla.arg = inla.arg, num.threads = 
##    num.threads, ", " blas.num.threads = blas.num.threads, keep = keep, 
##    working.directory = working.directory, ", " silent = silent, inla.mode 
##    = inla.mode, safe = FALSE, debug = debug, ", " .parent.frame = 
##    .parent.frame)") 
## Time used:
##     Pre = 1.37, Running = 0.437, Post = 0.185, Total = 1.99 
## Fixed effects:
##               mean    sd 0.025quant 0.5quant 0.975quant   mode kld
## (Intercept)  0.430 0.176      0.085    0.430      0.775  0.430   0
## pov          0.274 0.028      0.218    0.274      0.329  0.274   0
## wrk         -0.082 0.028     -0.136   -0.082     -0.027 -0.082   0
## poc2        -0.051 0.010     -0.070   -0.051     -0.031 -0.051   0
## ruralflag   -0.079 0.045     -0.168   -0.079      0.010 -0.079   0
## hcsa         0.143 0.036      0.073    0.143      0.214  0.143   0
## 
## Random effects:
##   Name     Model
##     re_u Besags ICAR model
##    re_v IID model
## 
## Model hyperparameters:
##                        mean       sd 0.025quant 0.5quant 0.975quant    mode
## Precision for re_u     3.45 2.26e-01       3.02     3.45       3.91    3.45
## Precision for re_v 17903.05 1.80e+04    1160.65 12410.48   66053.15 3157.07
## 
## Deviance Information Criterion (DIC) ...............: 5806.09
## Deviance Information Criterion (DIC, saturated) ....: 1097.57
## Effective number of parameters .....................: 526.02
## 
## Watanabe-Akaike information criterion (WAIC) ...: 5685.91
## Effective number of parameters .................: 291.81
## 
## Marginal log-Likelihood:  -4219.10 
## CPO, PIT is computed 
## Posterior summaries for the linear predictor and the fitted values are computed
## (Posterior marginals needs also 'control.compute=list(return.marginals.predictor=TRUE)')
```

exponentiate parameters

```
fixed <- exp(res$summary.fixed[-1, c(3,4,5)])
fixed
```

```
##           0.025quant  0.5quant 0.975quant
## pov        1.2432744 1.3146956  1.3900351
## wrk        0.8727653 0.9215162  0.9729108
## poc2       0.9321045 0.9507122  0.9696889
## ruralflag  0.8454782 0.9243493  1.0104766
## hcsa       1.0751956 1.1538537  1.2382665
```

Plot the posterior distribution of the percent of adults living below
FPL coefficient.

```
library(ggplot2)
marginal <- inla.smarginal(res$marginals.fixed$pov)
marginal <- data.frame(marginal)
ggplot(marginal, aes(x = x, y = y)) + geom_line() + 
  labs(x = expression(beta[1]), y = "Density") + 
  geom_vline(xintercept = 0, col = "blue") + theme_bw()
```

Working age plot

```
marginal <- inla.smarginal(res$marginals.fixed$wrk)
marginal <- data.frame(marginal)
ggplot(marginal, aes(x = x, y = y)) + geom_line() + 
  labs(x = expression(beta[1]), y = "Density") + 
  geom_vline(xintercept = 0, col = "blue") + theme_bw()
```

Identifying as a race/ethnicity other than non-Hispanic white

```
marginal <- inla.smarginal(res$marginals.fixed$poc2)
marginal <- data.frame(marginal)
ggplot(marginal, aes(x = x, y = y)) + geom_line() + 
  labs(x = expression(beta[1]), y = "Density") + 
  geom_vline(xintercept = 0, col = "blue") + theme_bw()
```

Rural

```
marginal <- inla.smarginal(res$marginals.fixed$ruralflag)
marginal <- data.frame(marginal)
ggplot(marginal, aes(x = x, y = y)) + geom_line() + 
  labs(x = expression(beta[1]), y = "Density") + 
  geom_vline(xintercept = 0, col = "blue") + theme_bw()
```

HCSA

```
marginal <- inla.smarginal(res$marginals.fixed$hcsa)
marginal <- data.frame(marginal)
ggplot(marginal, aes(x = x, y = y)) + geom_line() + 
  labs(x = expression(beta[1]), y = "Density") + 
  geom_vline(xintercept = 0, col = "blue") + theme_bw()
```

### 6.5 Sensitivity Analysis

Conduct a sensitivity analysis on the prior in the mode. Will compare
the default priors with priors defined by Virgilio Gómez-Rubioin
Bayesian inference with INLA available at https://becarioprecario.bitbucket.io/inla-gitbook/ch-priors.html#sec:pcpriors

```
# half-normal
HN.prior = "expression: 
  tau0 = 0.001;
  sigma = exp(-theta/2);
  log_dens = log(2) - 0.5 * log(2 * pi) + 0.5 * log(tau0);
  log_dens = log_dens - 0.5 * tau0 * sigma^2;
  log_dens = log_dens - log(2) - theta / 2;
  return(log_dens);  
"

# half-cauchy
HC.prior  = "expression:
  sigma = exp(-theta/2);
  gamma = 25;
  log_dens = log(2) - log(pi) - log(gamma);
  log_dens = log_dens - log(1 + (sigma / gamma)^2);
  log_dens = log_dens - log(2) - theta / 2;
  return(log_dens);
"

# half-t
HT.prior = "expression:
  sigma = exp(-theta/2);
  nu = 3;
  log_dens = 0 - 0.5 * log(nu * pi) - (-0.1207822);
  log_dens = log_dens - 0.5 * (nu + 1) * log(1 + sigma * sigma);
  log_dens = log_dens - log(2) - theta / 2;
  return(log_dens);
"

# uniform
UN.prior = "expression:
  log_dens = 0 - log(2) - theta / 2;
  return(log_dens);
"

# create prior list to loop through, will also evaluate penalized complexity prior
prior.list = list(
  default = list(prec = list(prior = "loggamma", param = c(1, 0.00005))),
  half.normal = list(prec = list(prior = HN.prior)),
  half.cauchy = list(prec = list(prior = HC.prior)),
  h.t = list(prec = list(prior = HT.prior)),
  uniform = list(prec = list(prior = UN.prior)),
  pc.prec = list(prec = list(prior = "pc.prec", param = c(5, 0.01)))
) 

# rerun the model using lapply()
set.seed(0111022)
map.models <- lapply(prior.list, function(tau.prior) {
  inla(cases ~ 1 + pctpov + wrkage + poc + ruralflag + hcsa + 
         f(re_u, model = "bym", graph = g, scale.model = TRUE) + 
                        f(re_v, model = "iid", hyper = tau.prior), 
       family = "poisson", 
       data = map.inla, E = E,
       control.compute = list(dic = TRUE, waic = TRUE, cpo = TRUE,
                                   return.marginals.predictor = TRUE),
       control.predictor = list(compute = TRUE))
})

summary(map.models$default)
```

```
## 
## Call:
##    c("inla.core(formula = formula, family = family, contrasts = contrasts, 
##    ", " data = data, quantiles = quantiles, E = E, offset = offset, ", " 
##    scale = scale, weights = weights, Ntrials = Ntrials, strata = strata, 
##    ", " lp.scale = lp.scale, link.covariates = link.covariates, verbose = 
##    verbose, ", " lincomb = lincomb, selection = selection, control.compute 
##    = control.compute, ", " control.predictor = control.predictor, 
##    control.family = control.family, ", " control.inla = control.inla, 
##    control.fixed = control.fixed, ", " control.mode = control.mode, 
##    control.expert = control.expert, ", " control.hazard = control.hazard, 
##    control.lincomb = control.lincomb, ", " control.update = 
##    control.update, control.lp.scale = control.lp.scale, ", " 
##    control.pardiso = control.pardiso, only.hyperparam = only.hyperparam, 
##    ", " inla.call = inla.call, inla.arg = inla.arg, num.threads = 
##    num.threads, ", " blas.num.threads = blas.num.threads, keep = keep, 
##    working.directory = working.directory, ", " silent = silent, inla.mode 
##    = inla.mode, safe = FALSE, debug = debug, ", " .parent.frame = 
##    .parent.frame)") 
## Time used:
##     Pre = 1.25, Running = 0.404, Post = 0.0228, Total = 1.67 
## Fixed effects:
##               mean    sd 0.025quant 0.5quant 0.975quant   mode kld
## (Intercept)  0.432 0.176      0.086    0.431      0.778  0.431   0
## pctpov       0.027 0.003      0.022    0.027      0.033  0.027   0
## wrkage      -0.008 0.003     -0.014   -0.008     -0.003 -0.008   0
## poc         -0.005 0.001     -0.007   -0.005     -0.003 -0.005   0
## ruralflag   -0.079 0.046     -0.168   -0.079      0.011 -0.079   0
## hcsa         0.143 0.036      0.072    0.143      0.214  0.143   0
## 
## Random effects:
##   Name     Model
##     re_u BYM model
##    re_v IID model
## 
## Model hyperparameters:
##                                            mean       sd 0.025quant 0.5quant
## Precision for re_u (iid component)      1639.88 2.49e+03      86.25   891.63
## Precision for re_u (spatial component)     3.50 2.42e-01       3.04     3.50
## Precision for re_v                     15937.19 1.67e+04    1014.24 10771.02
##                                        0.975quant    mode
## Precision for re_u (iid component)        7850.19  222.85
## Precision for re_u (spatial component)       4.00    3.50
## Precision for re_v                       60783.40 2755.31
## 
## Deviance Information Criterion (DIC) ...............: 5806.53
## Deviance Information Criterion (DIC, saturated) ....: 1098.02
## Effective number of parameters .....................: 526.23
## 
## Watanabe-Akaike information criterion (WAIC) ...: 5686.53
## Effective number of parameters .................: 292.07
## 
## Marginal log-Likelihood:  -3726.91 
## CPO, PIT is computed 
## Posterior summaries for the linear predictor and the fitted values are computed
## (Posterior marginals needs also 'control.compute=list(return.marginals.predictor=TRUE)')
```

```
summary(map.models$half.normal)
```

```
## 
## Call:
##    c("inla.core(formula = formula, family = family, contrasts = contrasts, 
##    ", " data = data, quantiles = quantiles, E = E, offset = offset, ", " 
##    scale = scale, weights = weights, Ntrials = Ntrials, strata = strata, 
##    ", " lp.scale = lp.scale, link.covariates = link.covariates, verbose = 
##    verbose, ", " lincomb = lincomb, selection = selection, control.compute 
##    = control.compute, ", " control.predictor = control.predictor, 
##    control.family = control.family, ", " control.inla = control.inla, 
##    control.fixed = control.fixed, ", " control.mode = control.mode, 
##    control.expert = control.expert, ", " control.hazard = control.hazard, 
##    control.lincomb = control.lincomb, ", " control.update = 
##    control.update, control.lp.scale = control.lp.scale, ", " 
##    control.pardiso = control.pardiso, only.hyperparam = only.hyperparam, 
##    ", " inla.call = inla.call, inla.arg = inla.arg, num.threads = 
##    num.threads, ", " blas.num.threads = blas.num.threads, keep = keep, 
##    working.directory = working.directory, ", " silent = silent, inla.mode 
##    = inla.mode, safe = FALSE, debug = debug, ", " .parent.frame = 
##    .parent.frame)") 
## Time used:
##     Pre = 1.24, Running = 0.433, Post = 0.0242, Total = 1.7 
## Fixed effects:
##               mean    sd 0.025quant 0.5quant 0.975quant   mode kld
## (Intercept)  0.447 0.181      0.093    0.447      0.802  0.447   0
## pctpov       0.027 0.003      0.021    0.027      0.033  0.027   0
## wrkage      -0.009 0.003     -0.014   -0.009     -0.003 -0.009   0
## poc         -0.005 0.001     -0.007   -0.005     -0.003 -0.005   0
## ruralflag   -0.077 0.046     -0.168   -0.077      0.014 -0.077   0
## hcsa         0.146 0.037      0.073    0.145      0.218  0.145   0
## 
## Random effects:
##   Name     Model
##     re_u BYM model
##    re_v IID model
## 
## Model hyperparameters:
##                                           mean       sd 0.025quant 0.5quant
## Precision for re_u (iid component)     1899.08 1828.190     161.05  1359.37
## Precision for re_u (spatial component)    3.99    0.431       3.13     4.00
## Precision for re_v                      102.25   59.432      39.32    86.64
##                                        0.975quant   mode
## Precision for re_u (iid component)        6782.04 459.30
## Precision for re_u (spatial component)       4.81   4.08
## Precision for re_v                         261.57  63.45
## 
## Deviance Information Criterion (DIC) ...............: 5806.74
## Deviance Information Criterion (DIC, saturated) ....: 1098.22
## Effective number of parameters .....................: 526.77
## 
## Watanabe-Akaike information criterion (WAIC) ...: 5685.77
## Effective number of parameters .................: 291.77
## 
## Marginal log-Likelihood:  -3732.13 
## CPO, PIT is computed 
## Posterior summaries for the linear predictor and the fitted values are computed
## (Posterior marginals needs also 'control.compute=list(return.marginals.predictor=TRUE)')
```

```
summary(map.models$half.cauchy)
```

```
## 
## Call:
##    c("inla.core(formula = formula, family = family, contrasts = contrasts, 
##    ", " data = data, quantiles = quantiles, E = E, offset = offset, ", " 
##    scale = scale, weights = weights, Ntrials = Ntrials, strata = strata, 
##    ", " lp.scale = lp.scale, link.covariates = link.covariates, verbose = 
##    verbose, ", " lincomb = lincomb, selection = selection, control.compute 
##    = control.compute, ", " control.predictor = control.predictor, 
##    control.family = control.family, ", " control.inla = control.inla, 
##    control.fixed = control.fixed, ", " control.mode = control.mode, 
##    control.expert = control.expert, ", " control.hazard = control.hazard, 
##    control.lincomb = control.lincomb, ", " control.update = 
##    control.update, control.lp.scale = control.lp.scale, ", " 
##    control.pardiso = control.pardiso, only.hyperparam = only.hyperparam, 
##    ", " inla.call = inla.call, inla.arg = inla.arg, num.threads = 
##    num.threads, ", " blas.num.threads = blas.num.threads, keep = keep, 
##    working.directory = working.directory, ", " silent = silent, inla.mode 
##    = inla.mode, safe = FALSE, debug = debug, ", " .parent.frame = 
##    .parent.frame)") 
## Time used:
##     Pre = 1.28, Running = 0.432, Post = 0.0232, Total = 1.73 
## Fixed effects:
##               mean    sd 0.025quant 0.5quant 0.975quant   mode kld
## (Intercept)  0.447 0.181      0.093    0.447      0.802  0.447   0
## pctpov       0.027 0.003      0.021    0.027      0.033  0.027   0
## wrkage      -0.009 0.003     -0.014   -0.009     -0.003 -0.009   0
## poc         -0.005 0.001     -0.007   -0.005     -0.003 -0.005   0
## ruralflag   -0.077 0.046     -0.168   -0.077      0.014 -0.077   0
## hcsa         0.146 0.037      0.073    0.145      0.218  0.145   0
## 
## Random effects:
##   Name     Model
##     re_u BYM model
##    re_v IID model
## 
## Model hyperparameters:
##                                           mean       sd 0.025quant 0.5quant
## Precision for re_u (iid component)     1898.75 1827.952     161.02  1359.10
## Precision for re_u (spatial component)    3.99    0.431       3.13     4.00
## Precision for re_v                      102.27   59.452      39.33    86.66
##                                        0.975quant   mode
## Precision for re_u (iid component)        6781.12 459.21
## Precision for re_u (spatial component)       4.81   4.08
## Precision for re_v                         261.65  63.46
## 
## Deviance Information Criterion (DIC) ...............: 5806.74
## Deviance Information Criterion (DIC, saturated) ....: 1098.22
## Effective number of parameters .....................: 526.77
## 
## Watanabe-Akaike information criterion (WAIC) ...: 5685.77
## Effective number of parameters .................: 291.77
## 
## Marginal log-Likelihood:  -3732.12 
## CPO, PIT is computed 
## Posterior summaries for the linear predictor and the fitted values are computed
## (Posterior marginals needs also 'control.compute=list(return.marginals.predictor=TRUE)')
```

```
summary(map.models$h.t)
```

```
## 
## Call:
##    c("inla.core(formula = formula, family = family, contrasts = contrasts, 
##    ", " data = data, quantiles = quantiles, E = E, offset = offset, ", " 
##    scale = scale, weights = weights, Ntrials = Ntrials, strata = strata, 
##    ", " lp.scale = lp.scale, link.covariates = link.covariates, verbose = 
##    verbose, ", " lincomb = lincomb, selection = selection, control.compute 
##    = control.compute, ", " control.predictor = control.predictor, 
##    control.family = control.family, ", " control.inla = control.inla, 
##    control.fixed = control.fixed, ", " control.mode = control.mode, 
##    control.expert = control.expert, ", " control.hazard = control.hazard, 
##    control.lincomb = control.lincomb, ", " control.update = 
##    control.update, control.lp.scale = control.lp.scale, ", " 
##    control.pardiso = control.pardiso, only.hyperparam = only.hyperparam, 
##    ", " inla.call = inla.call, inla.arg = inla.arg, num.threads = 
##    num.threads, ", " blas.num.threads = blas.num.threads, keep = keep, 
##    working.directory = working.directory, ", " silent = silent, inla.mode 
##    = inla.mode, safe = FALSE, debug = debug, ", " .parent.frame = 
##    .parent.frame)") 
## Time used:
##     Pre = 1.24, Running = 0.529, Post = 0.0226, Total = 1.79 
## Fixed effects:
##               mean    sd 0.025quant 0.5quant 0.975quant   mode kld
## (Intercept)  0.447 0.181      0.093    0.447      0.802  0.447   0
## pctpov       0.027 0.003      0.021    0.027      0.033  0.027   0
## wrkage      -0.009 0.003     -0.014   -0.009     -0.003 -0.009   0
## poc         -0.005 0.001     -0.007   -0.005     -0.003 -0.005   0
## ruralflag   -0.077 0.046     -0.168   -0.077      0.014 -0.077   0
## hcsa         0.145 0.037      0.073    0.145      0.218  0.145   0
## 
## Random effects:
##   Name     Model
##     re_u BYM model
##    re_v IID model
## 
## Model hyperparameters:
##                                           mean       sd 0.025quant 0.5quant
## Precision for re_u (iid component)     1929.42 1873.611     159.20  1375.30
## Precision for re_u (spatial component)    3.99    0.432       3.12     4.00
## Precision for re_v                      104.48   61.108      40.27    88.44
##                                        0.975quant   mode
## Precision for re_u (iid component)        6928.69 451.74
## Precision for re_u (spatial component)       4.81   4.10
## Precision for re_v                         268.87  64.32
## 
## Deviance Information Criterion (DIC) ...............: 5806.78
## Deviance Information Criterion (DIC, saturated) ....: 1098.27
## Effective number of parameters .....................: 526.71
## 
## Watanabe-Akaike information criterion (WAIC) ...: 5685.97
## Effective number of parameters .................: 291.84
## 
## Marginal log-Likelihood:  -3729.47 
## CPO, PIT is computed 
## Posterior summaries for the linear predictor and the fitted values are computed
## (Posterior marginals needs also 'control.compute=list(return.marginals.predictor=TRUE)')
```

```
summary(map.models$uniform)
```

```
## 
## Call:
##    c("inla.core(formula = formula, family = family, contrasts = contrasts, 
##    ", " data = data, quantiles = quantiles, E = E, offset = offset, ", " 
##    scale = scale, weights = weights, Ntrials = Ntrials, strata = strata, 
##    ", " lp.scale = lp.scale, link.covariates = link.covariates, verbose = 
##    verbose, ", " lincomb = lincomb, selection = selection, control.compute 
##    = control.compute, ", " control.predictor = control.predictor, 
##    control.family = control.family, ", " control.inla = control.inla, 
##    control.fixed = control.fixed, ", " control.mode = control.mode, 
##    control.expert = control.expert, ", " control.hazard = control.hazard, 
##    control.lincomb = control.lincomb, ", " control.update = 
##    control.update, control.lp.scale = control.lp.scale, ", " 
##    control.pardiso = control.pardiso, only.hyperparam = only.hyperparam, 
##    ", " inla.call = inla.call, inla.arg = inla.arg, num.threads = 
##    num.threads, ", " blas.num.threads = blas.num.threads, keep = keep, 
##    working.directory = working.directory, ", " silent = silent, inla.mode 
##    = inla.mode, safe = FALSE, debug = debug, ", " .parent.frame = 
##    .parent.frame)") 
## Time used:
##     Pre = 1.24, Running = 0.414, Post = 0.023, Total = 1.68 
## Fixed effects:
##               mean    sd 0.025quant 0.5quant 0.975quant   mode kld
## (Intercept)  0.447 0.181      0.093    0.447      0.802  0.447   0
## pctpov       0.027 0.003      0.021    0.027      0.033  0.027   0
## wrkage      -0.009 0.003     -0.014   -0.009     -0.003 -0.009   0
## poc         -0.005 0.001     -0.007   -0.005     -0.003 -0.005   0
## ruralflag   -0.077 0.046     -0.168   -0.077      0.014 -0.077   0
## hcsa         0.146 0.037      0.073    0.145      0.218  0.145   0
## 
## Random effects:
##   Name     Model
##     re_u BYM model
##    re_v IID model
## 
## Model hyperparameters:
##                                           mean       sd 0.025quant 0.5quant
## Precision for re_u (iid component)     1899.85 1828.394     161.28  1360.17
## Precision for re_u (spatial component)    3.99    0.431       3.13     4.00
## Precision for re_v                      102.16   59.341      39.30    86.58
##                                        0.975quant   mode
## Precision for re_u (iid component)        6783.29 459.98
## Precision for re_u (spatial component)       4.81   4.08
## Precision for re_v                         261.22  63.44
## 
## Deviance Information Criterion (DIC) ...............: 5806.73
## Deviance Information Criterion (DIC, saturated) ....: 1098.22
## Effective number of parameters .....................: 526.77
## 
## Watanabe-Akaike information criterion (WAIC) ...: 5685.76
## Effective number of parameters .................: 291.77
## 
## Marginal log-Likelihood:  -3728.46 
## CPO, PIT is computed 
## Posterior summaries for the linear predictor and the fitted values are computed
## (Posterior marginals needs also 'control.compute=list(return.marginals.predictor=TRUE)')
```

```
summary(map.models$pc.prec)
```

```
## 
## Call:
##    c("inla.core(formula = formula, family = family, contrasts = contrasts, 
##    ", " data = data, quantiles = quantiles, E = E, offset = offset, ", " 
##    scale = scale, weights = weights, Ntrials = Ntrials, strata = strata, 
##    ", " lp.scale = lp.scale, link.covariates = link.covariates, verbose = 
##    verbose, ", " lincomb = lincomb, selection = selection, control.compute 
##    = control.compute, ", " control.predictor = control.predictor, 
##    control.family = control.family, ", " control.inla = control.inla, 
##    control.fixed = control.fixed, ", " control.mode = control.mode, 
##    control.expert = control.expert, ", " control.hazard = control.hazard, 
##    control.lincomb = control.lincomb, ", " control.update = 
##    control.update, control.lp.scale = control.lp.scale, ", " 
##    control.pardiso = control.pardiso, only.hyperparam = only.hyperparam, 
##    ", " inla.call = inla.call, inla.arg = inla.arg, num.threads = 
##    num.threads, ", " blas.num.threads = blas.num.threads, keep = keep, 
##    working.directory = working.directory, ", " silent = silent, inla.mode 
##    = inla.mode, safe = FALSE, debug = debug, ", " .parent.frame = 
##    .parent.frame)") 
## Time used:
##     Pre = 1.32, Running = 0.489, Post = 0.0243, Total = 1.83 
## Fixed effects:
##               mean    sd 0.025quant 0.5quant 0.975quant   mode kld
## (Intercept)  0.446 0.180      0.094    0.446      0.800  0.446   0
## pctpov       0.027 0.003      0.021    0.027      0.033  0.027   0
## wrkage      -0.009 0.003     -0.014   -0.009     -0.003 -0.009   0
## poc         -0.005 0.001     -0.007   -0.005     -0.003 -0.005   0
## ruralflag   -0.077 0.046     -0.168   -0.077      0.014 -0.077   0
## hcsa         0.145 0.037      0.073    0.145      0.218  0.145   0
## 
## Random effects:
##   Name     Model
##     re_u BYM model
##    re_v IID model
## 
## Model hyperparameters:
##                                           mean       sd 0.025quant 0.5quant
## Precision for re_u (iid component)     1898.12 1833.540     152.27  1355.32
## Precision for re_u (spatial component)    4.00    0.415       3.14     4.02
## Precision for re_v                      111.66   65.895      44.31    94.83
##                                        0.975quant   mode
## Precision for re_u (iid component)        6788.27 431.52
## Precision for re_u (spatial component)       4.76   4.16
## Precision for re_v                         291.72  67.79
## 
## Deviance Information Criterion (DIC) ...............: 5806.99
## Deviance Information Criterion (DIC, saturated) ....: 1098.48
## Effective number of parameters .....................: 526.59
## 
## Watanabe-Akaike information criterion (WAIC) ...: 5686.59
## Effective number of parameters .................: 292.05
## 
## Marginal log-Likelihood:  -3728.47 
## CPO, PIT is computed 
## Posterior summaries for the linear predictor and the fitted values are computed
## (Posterior marginals needs also 'control.compute=list(return.marginals.predictor=TRUE)')
```

No differences in beta coefficients or fit parameters identified,
results remain unchanged.

We’ll also evaluate our residuals to test whether there is
correlation in our error terms. There are not pearson’s residuals for
INLA models, so will calculate them manually.

```
pred.mean <- map.inla$E*res$summary.fitted.values$mean
pred.sd <- map.inla$E*res$summary.fitted.values$sd
pearson.residuals <- (map.inla$cases - pred.mean)/pred.sd

plot(res)
```

```
inla.dev.new()
hist(pearson.residuals, prob = TRUE, n = 100)
```

Also evaluate PIT (probability integral transform) values.  
(*PIT is the probability of a new response less than the observed
response using a model based on the rest of the data. Expect PIT values
to be uniformly distributed if the model assumptions are
correct)*

```
plot(res, plot.fixed.effects=FALSE, plot.lincomb=FALSE, plot.random.effects=FALSE,
     plot.hyperparameters=FALSE, plot.predictor=FALSE, plot.q=FALSE, plot.cpo=TRUE,
     single=FALSE)
```

Several observations have CPO low values, indicating we have some
problems with our model. Most likely, there are several covariates
missing that explain the response.

### 6.6 Add Results to Data for Mapping

```
head(res$summary.fitted.values)
```

```
##                           mean         sd 0.025quant  0.5quant 0.975quant
## fitted.Predictor.001 1.6168461 0.17527271  1.3004265 1.6074143  1.9870119
## fitted.Predictor.002 0.8151882 0.05318473  0.7158561 0.8134594  0.9243649
## fitted.Predictor.003 1.3240836 0.03339364  1.2597966 1.3236627  1.3907667
## fitted.Predictor.004 1.5052729 0.19882829  1.1529779 1.4923313  1.9313580
## fitted.Predictor.005 0.6863757 0.01616739  0.6552128 0.6861853  0.7186219
## fitted.Predictor.006 1.4185625 0.05000115  1.3230225 1.4176821  1.5191144
##                           mode
## fitted.Predictor.001 1.5887281
## fitted.Predictor.002 0.8100104
## fitted.Predictor.003 1.3228184
## fitted.Predictor.004 1.4668055
## fitted.Predictor.005 0.6858035
## fitted.Predictor.006 1.4159188
```

```
map.inla$RR <- res$summary.fitted.values[, "mean"]
map.inla$LL <- res$summary.fitted.values[, "0.025quant"]
map.inla$UL <- res$summary.fitted.values[, "0.975quant"]
```

# 7. Mapping Infection Risk

Map estimated infection risk on an interactive map that will show
labels when you hover over the MSSA to convey observed and expected
counts, percent of adults living below the federal poverty limit, RRs,
and lower/upper limits of 95% credible intervals.

```
l <- leaflet(map) %>% addTiles()

pal <- colorNumeric(palette = "YlOrRd", domain = map$SIR)

labels <- sprintf("<strong>%s</strong><br/>Observed: %s <br/>Expected: %s<br/> SIR: %s",
                  map$mssa, map$cases, round(map$E, 2), round(map$SIR, 2)) %>%
  lapply(htmltools::HTML)

l %>% addPolygons(color = "grey", weight = 1, fillColor = ~pal(SIR), fillOpacity = 0.5,
                  highlightOptions = highlightOptions(weight = 4),
                  label = labels,
                  labelOptions = labelOptions(style = list("font-weight" = "normal",
                                                           padding = "3px 8px"),
                                              textsize = "15px", direction = "auto")) %>%
  addLegend(pal = pal, values = ~SIR, opacity = 0.5, title = "SIR", position = "bottomright")
```

```
pal.rr <- colorNumeric(palette = "YlOrRd", domain = map.inla$RR)

labels.rr <- sprintf("<strong> %s </strong> <br/> 
  Observed: %s <br/> Expected: %s <br/> 
  Percent working age adults (18-64 years): %s <br/>
  Percent identifying as race/ethnicity other than non-Hispanic white: %s <br/>
  Ruralality: %s <br/>
  Percent adults living below federal poverty level: %s <br/> 
  HCSA: %s <br/> RR: %s (%s, %s)", 
                     map.inla$mssa, map.inla$cases, round(map.inla$E, 2),
                     round(map.inla$wrkage, 2), round(map.inla$poc, 2), map.inla$rural,
                     round(map.inla$pctpov, 2), map.inla$hcsa, round(map.inla$RR, 2), 
                     round(map.inla$LL, 2), round(map.inla$UL, 2)) %>%
  lapply(htmltools::HTML)


leaflet(map.inla) %>% addTiles() %>%
  addPolygons(color = "grey", weight = 1, fillColor = ~pal.rr(RR), 
              fillOpacity = 0.5, 
              highlightOptions = highlightOptions(weight = 4), 
              label = labels.rr, 
              labelOptions = labelOptions(
                style = list("font-weight" = "normal",
                             padding = "3px 8px"),
                textsize = "15px", direction = "auto")) %>%
  addLegend(pal = pal.rr, values = ~RR, opacity = 0.5, title = "RR", position = "bottomright")
```

```
# static map of RR
rr <- tm_shape(map.inla) +
  tm_polygons(c("RR"), style = "jenks",
              palette = "Reds",
              border.alpha = 0.1,
              title = "RR",
              legend.format = list(fun = function(x) paste0(formatC(x, digits = 2, 
                                                                    format = "f")))) +
  tm_layout(frame = FALSE)

tmap_save(rr, "figure4.png", width = 800, dpi = 300)
```

```
## Map saved to figure4.png
```

```
## Resolution: 800 by 926.76 pixels
```

```
## Size: 2.666667 by 3.0892 inches (300 dpi)
```

```
rr
```

```
# MSSAs with highest RR of CA-MRSA
highRR <- map.inla %>%
  select(mssa, RR, LL, UL) %>%
  arrange(desc(RR))

head(highRR, 10)
```

```
## Simple feature collection with 10 features and 4 fields
## Geometry type: GEOMETRY
## Dimension:     XY
## Bounding box:  xmin: -124.2558 ymin: 34.07928 xmax: -114.1312 ymax: 42.00105
## Geodetic CRS:  WGS 84
## # A tibble: 10 × 5
## # Rowwise: 
##    mssa                                 RR    LL    UL                  geometry
##    <chr>                             <dbl> <dbl> <dbl>            <GEOMETRY [°]>
##  1 Clearlake/Clearlake Oaks           5.93  5.69  6.17 POLYGON ((-122.6148 38.9…
##  2 Lucerne/Nice/Upper Lake            4.73  4.43  5.04 POLYGON ((-122.8339 39.0…
##  3 Dunsmuir                           4.36  3.75  5.04 MULTIPOLYGON (((-122.172…
##  4 Alta Sierra/Bodfish/Glennville/K…  3.85  3.63  4.08 POLYGON ((-118.4127 35.4…
##  5 Crescent City/Gasquet/Klamath/Sm…  3.73  3.57  3.91 POLYGON ((-124.1842 41.7…
##  6 Bailey Creek/Canyondam/Chester/E…  3.65  3.23  4.10 POLYGON ((-121.03 40.234…
##  7 Garberville/Redway                 3.63  3.34  3.94 POLYGON ((-123.7855 40.0…
##  8 Brooktrails/Pine Mountain/Willits  3.58  3.36  3.82 POLYGON ((-123.3446 39.6…
##  9 Ukiah                              3.55  3.40  3.71 POLYGON ((-123.1991 39.1…
## 10 Big River/Needles                  3.52  3.20  3.85 POLYGON ((-114.229 34.18…
```

```
# MSSAs with lowest RR of CA-MRSA
lowRR <- map.inla %>%
  select(mssa, RR, LL, UL) %>%
  arrange(RR)

head(lowRR, 10)
```

```
## Simple feature collection with 10 features and 4 fields
## Geometry type: GEOMETRY
## Dimension:     XY
## Bounding box:  xmin: -122.5132 ymin: 32.87517 xmax: -117.0556 ymax: 37.90604
## Geodetic CRS:  WGS 84
## # A tibble: 10 × 5
## # Rowwise: 
##    mssa                                 RR    LL    UL                  geometry
##    <chr>                             <dbl> <dbl> <dbl>            <GEOMETRY [°]>
##  1 Mammoth Lakes                     0.144 0.101 0.197 MULTIPOLYGON (((-118.917…
##  2 Carmel Valley/Del Dios/Escondido… 0.313 0.296 0.332 POLYGON ((-117.1678 32.9…
##  3 Foothill Ranch/IrvineNortheast/L… 0.315 0.296 0.334 POLYGON ((-117.7758 33.7…
##  4 Cupertino/Rancho Rinconada/San J… 0.335 0.312 0.358 POLYGON ((-122.0026 37.2…
##  5 Mira Mesa/Scripps Miramar Ranch   0.351 0.329 0.373 POLYGON ((-117.1347 32.8…
##  6 Sunnyvale South                   0.382 0.356 0.408 POLYGON ((-122.051 37.33…
##  7 Irvine Central/Tustin East        0.385 0.360 0.411 POLYGON ((-117.7952 33.6…
##  8 Alamo/Danville/Diablo/San Ramon   0.404 0.382 0.427 POLYGON ((-121.9403 37.7…
##  9 Coto de Caza/Las Flores/Mission … 0.408 0.386 0.430 POLYGON ((-117.6501 33.5…
## 10 Golden Gate Park/Parkside/Sunset… 0.410 0.382 0.439 POLYGON ((-122.4769 37.7…
```
